# Alterations of NK Cell Phenotype in the Disease Course of Multiple Myeloma

**DOI:** 10.3390/cancers13020226

**Published:** 2021-01-10

**Authors:** Tatiana Pazina, Alexander W. MacFarlane, Luca Bernabei, Essel Dulaimi, Rebecca Kotcher, Clinton Yam, Natalie A. Bezman, Michael D. Robbins, Eric A. Ross, Kerry S. Campbell, Adam D. Cohen

**Affiliations:** 1 Blood Cell Development and Function Program, Fox Chase Cancer Center, Philadelphia, PA 19111, USA; tatiana.pazina@gmail.com (T.P.); alexander.macfarlane@fccc.edu (A.W.M.IV); 2 FSBSI “Institute of Experimental Medicine”, Department of General Pathology and Pathological Physiology, 197376 St. Petersburg, Russia; 3 Abramson Cancer Center, University of Pennsylvania, Philadelphia, PA 19104, USA; lubernabei@gmail.com (L.B.); Rebecca.Kotcher@pennmedicine.upenn.edu (R.K.); CYam@mdanderson.org (C.Y.); 4 Department of Pathology, Fox Chase Cancer Center, Philadelphia, PA 19111, USA; Edulaimi@korgene.com; 5 Bristol-Myers Squibb, Redwood City, CA 94063, USA; nbezman@arsenalbio.com; 6 Bristol-Myers Squibb, Cambridge, MA 02142, USA; mrobbinsct@gmail.com; 7 Biostatistics and Bioinformatics Facility, Fox Chase Cancer Center, Philadelphia, PA 19111, USA; Eric.Ross@fccc.edu

**Keywords:** multiple myeloma, NK cells, DNAM-1, NKG2D, NCR, SLAMF7, GITR, bone marrow, blood

## Abstract

**Simple Summary:**

Multiple myeloma (MM) is a deadly cancer localized in the bone marrow, where changes can support progression and therapy resistance. This study examined the expression of numerous biological markers on natural killer (NK) cells in blood and bone marrow of patients with MM. NK cells play key roles in the innate immunosurveillance of MM, so we sought to identify biomarkers on NK cells that may be prognostic for patient outcomes and identify new therapeutic targets in these patients. Biomarker expression was compared on NK cells between MM disease stages and healthy donors, between blood and bone marrow, and associations with disease progression. The study shows that loss of certain biomarkers on NK cells may limit their anti-tumor function in MM patients, that several drug-targetable biomarkers are upregulated on NK cells, and that high expression of the biomarker, SLAMF7, may have prognostic potential to identify patients more likely to show rapid disease progression.

**Abstract:**

Accumulating evidence demonstrates important roles for natural killer (NK) cells in controlling multiple myeloma (MM). A prospective flow cytometry-based analysis of NK cells in the blood and bone marrow (BM) of MM patient subgroups was performed (smoldering (SMM), newly diagnosed (ND), relapsed/refractory, (RR) and post-stem cell transplantation (pSCT)). Assessments included the biomarker expression and function of NK cells, correlations between the expression of receptors on NK cells with their ligands on myeloma cells, and comparisons between MM patient subgroups and healthy controls. The most striking differences from healthy controls were found in RR and pSCT patients, in which NK cells were less mature and expressed reduced levels of the activating receptors DNAM-1, NKG2D, and CD16. These differences were more pronounced in the BM than in blood, including upregulation of the therapeutic targets TIM3, TIGIT, ICOS, and GITR. Their expression suggests NK cells became exhausted upon chronic encounters with the tumor. A high expression of SLAMF7 on blood NK cells correlated with shorter progression-free survival. This correlation was particularly evident in ND patients, including on mature CD56^dim^ NK cells in the BM. Thus, our NK cell analysis identified possible therapeutic targets in MM and a biomarker with prognostic potential for disease progression.

## 1. Introduction

Natural killer (NK) cells constitute about 10–15% of lymphocytes in the peripheral blood of healthy humans and are an important component of the innate immune system. They can spontaneously recognize and kill certain virus-infected and malignant cells, while also providing a potent source of inflammatory cytokines, especially interferon (IFN)-γ and tumor necrosis factor (TNF)-α, and chemokines [1]. The two major subsets of NK cells in human blood are the mature CD56^dim^ NK cells, which are highly cytolytic and heavily localized in the blood, and immature CD56^bright^ cells (about 5–20% of total NK cells), which primarily elicit cytokine responses and are more prevalent in tissues and lymph nodes [2,3]. Common germline-encoded activating receptors on NK cells involved in anti-tumor responses include DNAM-1, NKG2D, CD16 (FcγRIIIa), and the natural cytotoxicity receptors (NCRs; NKp30, NKp44, and NKp46) [1,4,5].

Multiple myeloma (MM), a malignancy of bone marrow (BM) plasma cells, is the second most common hematologic malignancy, with over 32,000 new cases and almost 13,000 deaths predicted in the United States in 2020 [6]. While MM remains an incurable disease for the vast majority of patients, novel treatment regimens incorporating proteasome inhibitors (PIs), immunomodulatory drugs (IMiDs), and monoclonal antibodies (mAbs) have significantly improved clinical outcomes and patient survival. Importantly, all of these therapies act not just directly on the myeloma cell, but also indirectly via interactions with the patient immune system, including NK cells [7,8,9]. In particular, the mAbs used in MM, including the anti-SLAMF7 mAb elotuzumab, the anti-CD38 mAbs daratumumab and isatuximab, and the anti-B cell maturation antigen (BCMA) antibody–drug conjugate belantamab mafadotin, all work in part by promoting antibody-dependent cellular cytotoxicity (ADCC) mediated by NK cells [10,11,12,13]. NK cells may also contribute to long-term disease control following hematopoietic stem cell transplantation for MM, as demonstrated by the impact of the killer immunoglobulin-like receptor (KIR) haplotype on progression-free survival [14,15]. Finally, NK cells can directly mediate MM cell killing [16,17], and both endogenous and gene-modified NK cells are undergoing exploration as adoptive cellular therapy for MM [18,19,20].

Growing evidence indicates that NK cells and MM cells may be mutually immuno-edited over the course of the disease, both by the MM tumor microenvironment (TME) and anti-MM therapies [21,22,23,24,25,26]. For example, Carbone et al. found that BM-derived myeloma tumor cells from early clinical stage MM patients express low levels of the NK cell inhibitory ligand, MHC class I and high expression of the NKG2D ligands, MHC class I polypeptide–related sequence A (MICA) and MICB, but tumors from later-stage disease had the opposite expression pattern and were less susceptible to NK cell cytotoxicity [21]. Blocking antibodies were also used in this study to show that NKG2D and NCRs (natural cytotoxicity receptors) (NKp30, NKp44, and NKp46) were important activating receptors, triggering the NK cell response to early-stage tumors. The expression of these and other activating (e.g., DNAM-1) or inhibitory (e.g., KIR, NKG2A) receptors on NK cells, as well as their ligands on MM cells, may also be altered in MM patients, and vary significantly from patient to patient [9,23,27]. These changes may potentially impact the response to NK cell-dependent therapies. Thus, better characterization of NK cells in MM patients at different stages of their disease may provide insights that lead to optimization of the timing and/or choice of these therapies, or potential novel combination partners, ultimately leading to more personalized medicine.

In this study, a prospective, comprehensive immune phenotyping analysis by flow cytometry of freshly obtained NK cells from the peripheral blood and BM of MM patients and healthy donors was performed. Maturation state, expression of receptors, activation, and function of NK cells were assessed. These parameters on NK cells in the blood were compared to those in the TME of the bone marrow, as well as comparing NK cell status in different disease stages of MM patients with those in healthy controls. Although this is an observational study, the ultimate goals were to identify biomarkers of disease progression and potential therapeutic targets to improve NK cell responsiveness in MM patients.

## 2. Results

### 2.1. NK Cell Frequencies, Biomarker Phenotypes, and Function in Peripheral Blood Mononuclear Cells (PBMCs) of MM Patients

To improve understanding of NK cell status in MM patients, a comprehensive prospective flow cytometry-based analysis of the frequencies, phenotype, and function of NK cells on fresh PBMCs and BM leukocyte samples from MM patients and healthy control donors (HDs) was performed. Samples were obtained from 7 patients with smoldering myeloma (SMM; six blood and three BM samples), 17 patients with active disease that were newly diagnosed and untreated (ND; 15 blood and 13 BM samples), 23 relapsed/refractory patients (RR; 22 blood and 13 BM samples), 14 patients sampled about 100 days after high-dose melphalan and post-autologous stem cell transplant (pSCT; 13 blood and 11 BM samples), and 19 HDs of similar median age. Patient diagnostic characteristics and treatment histories are summarized in Table 1. The primary focus for this report was the analysis of NK cells using the antibody panel presented in Table S1. This panel also stained T and B cells, but these results will not be detailed here. Another panel was used to stain NK cell ligands on myeloma cells in BM, as shown in Table S2. It should be noted that the antibody staining panels were significantly expanded after the first 14 patients were analyzed, so some immune parameters (especially mean fluorescence intensity (MFI) values) from this early set of patients (three SMM, three ND, four RR, and four pSCT) were not available to be included in the final dataset.

**Figure 1:**
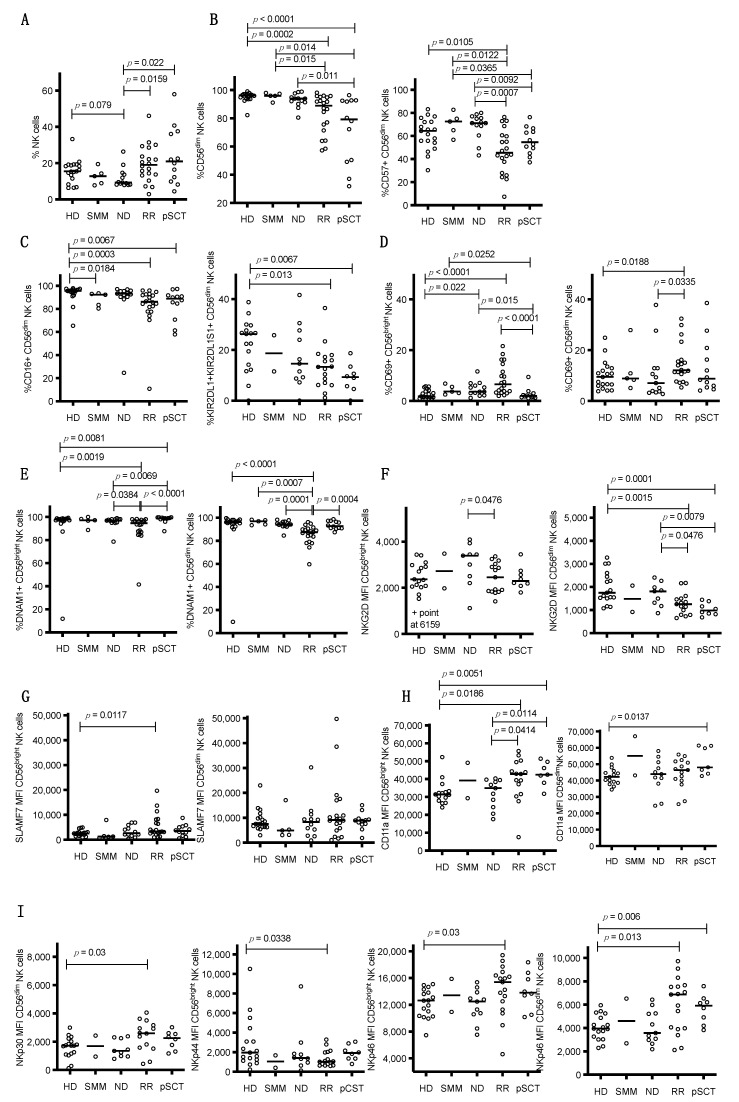
Percentages of various natural killer (NK) cell subsets and their expression of various NK cell markers in the blood of HDs and different MM patient subgroups. Each open circle designates a value for a distinct patient: HD = healthy donor, SMM = smoldering MM, ND = newly diagnosed, RR = relapsed/refractory, and pSCT = post-stem cell transplant, with horizontal lines marking medians. (**A**) The % of CD45^+^CD3^−^CD56^+^ NK cells in the viable lymphocyte gate. (**B**) % of total CD45^+^CD3^−^CD56^+^ NK cells that are CD56^dim^ (left) and % CD56^dim^ NK cells that are CD57^+^ (right), (**C**) % CD56^dim^ NK cells that are CD16^+^ (left) and % CD56^dim^ NK cells that are KIR2DS1^+^ and/or KIR2DL1^+^ (right), (**D**) % CD56^bright^ (left) and CD56^dim^ NK cells that are CD69^+^ (right), (**E**) % CD56^bright^ (left) and CD56^dim^ NK cells that are DNAM-1^+^ (right), (**F**) NKG2D expression level (NKG2D delta (d)MFI) on CD56^bright^ (left) and CD56^dim^ NK cells (right), (**G**) SLAMF7 expression level (SLAMF7 dMFI) on CD56^bright^ (left) and CD56^dim^ NK cells (right), (**H**) CD11a expression level (CD11a MFI) on CD56^bright^ (left) and CD56^dim^ NK cells (right), (**I**) natural cytotoxicity receptor (NCR) expression: NKp30 expression level (NKp30 dMFI) on CD56^dim^ NK cells, NKp46 expression level (NKp46 dMFI) on CD56^bright^ and CD56^dim^ NK cells, and NKp44 expression level (NKp44 MFI) on CD56^bright^ NK cells. Statistical comparisons between groups were calculated with an unpaired Wilcoxon rank-sum test.

The frequencies of NK cell populations in peripheral blood were analyzed using the gating strategy outlined in Figure S1A. Although not significantly different from control HDs, ND patients tended to have lower frequencies of NK cells as a percentage of total lymphocytes in peripheral blood, while RR and pSCT patients had significantly higher NK cell frequencies than those of ND patients, particularly in a subset of the RR and pSCT patients (Figure 1A). Compared to HDs, the SMM and ND patients had similar high percentages of mature cytolytic CD56^dim^ NK cells in peripheral blood, but RR and especially pSCT patients had significantly lower percentages of CD56^dim^ NK cells (Figure 1B, left panel). Comparatively, the RR and pSCT patients had higher percentages of immature, high cytokine producing/low cytolytic CD56^bright^ NK cells, which are the remaining NK cell population in our analysis [2]. The increased accumulation of total and immature CD56^bright^ NK cells in RR and pSCT patients may be a consequence of the active repopulation of the NK cell pool after depletion by previous therapies or high-dose chemotherapy and SCT. Consistent with this assumption, lower percentages of CD56^dim^ NK cells were found to express the terminal maturation marker CD57 (Figure 1B, right panel), FcγRIIIa (CD16) (Figure 1C, left panel), and a subset of killer cell Ig-like receptors (KIRs; KIR2DL1 and/or KIR2DS1) (Figure 1C, right panel) in both RR and pSCT patients, as compared to HDs. Expression of the activation marker, CD69, was also examined. As compared to HDs, CD69 expression was significantly elevated on both CD56^dim^ and CD56^bright^ NK cells of RR patients and on CD56^bright^ NK cells of ND patients (Figure 1D).

Next, the expression of a wide array of receptors on the surface of NK cells in peripheral blood was assessed. Representative staining profiles for these biomarkers are presented in Figure S1B. While the expression levels of most of these receptors did not differ significantly from that of NK cells in HDs, the expression levels of DNAX Accessory Molecule-1 (DNAM-1; CD226), NKG2D (CD314), SLAM family member 7 (SLAMF7; CD319), CD11a, and natural cytotoxicity receptors (NCRs) were found to be significantly altered in RR and pSCT patients. The percentage of NK cells expressing the activating receptor, DNAM-1, was significantly reduced in RR patients, particularly in the CD56^dim^ subset (Figure 1E). Similarly, the expression level (dMFI) of the NKG2D activating receptor was lower on CD56^dim^ NK cells from RR and pSCT patients (Figure 1F). Additionally, the expression level of the SLAMF7 costimulatory receptor was significantly increased on CD56^bright^ NK cells of RR patients, though with substantial inter-patient variability (Figure 1G). Furthermore, the expression level of CD11a (which heterodimerizes with CD18 to form the lymphocyte function-associated antigen 1 (LFA-1) adhesion molecule) was significantly higher on CD56^bright^ NK cells from RR patients and both CD56^dim^ and CD56^bright^ NK cells from pSCT patients (Figure 1H). Among the NCRs, RR patients exhibited an increased expression level of NKp30 (CD337) on CD56^dim^ NK cells. NKp46 (CD335) expression was also increased on both NK cell subsets, whereas the NKp44 (CD336) expression level was decreased on CD56^bright^ cells (Figure 1I). The NKp46 expression level was also increased on CD56^dim^ NK cells of pSCT patients (Figure 1I).

NK cell function was next assessed by comparing degranulation responses of NK cells from the peripheral blood of HD and MM patients. These experiments measured the expression of the degranulation marker CD107a on CD56^dim^ NK cells in PBMCs exposed to the MM.1R myeloma cell line in the absence or presence of the therapeutic anti-SLAMF7 antibody, elotuzumab. Elotuzumab stimulates antibody-dependent cellular cytotoxicity (ADCC) responses by NK cells and is used to treat RR MM patients in combination with lenalidomide or pomalidomide [10]. As shown in Figure S2, although some individual RR and pSCT patients exhibited lower NK cell expression of CD107a under ADCC conditions, none of the patient groups showed statistically significant differences in degranulation response, as compared to HDs, under any of the stimulation conditions. Thus, we conclude that the majority of MM patients tested have functionally competent NK cells capable of mediating ADCC response to myeloma tumor cells in the presence of elotuzumab, at least under the conditions tested.

### 2.2. Comparison of NK Cell Phenotype in BM and Blood of MM Patients

**Figure 2:**
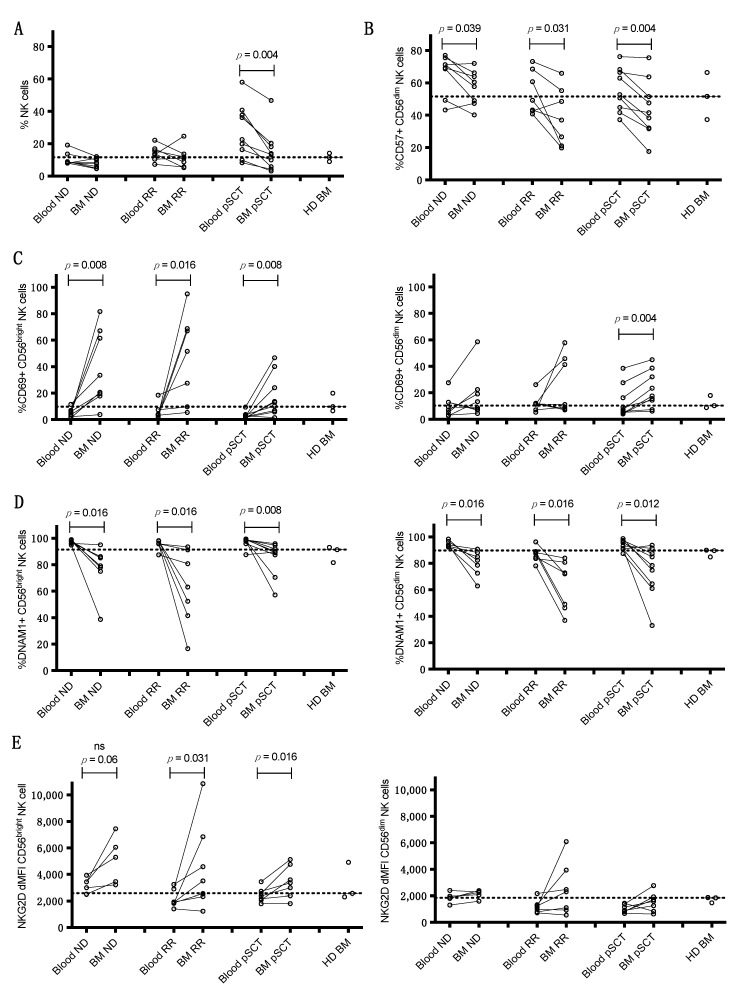

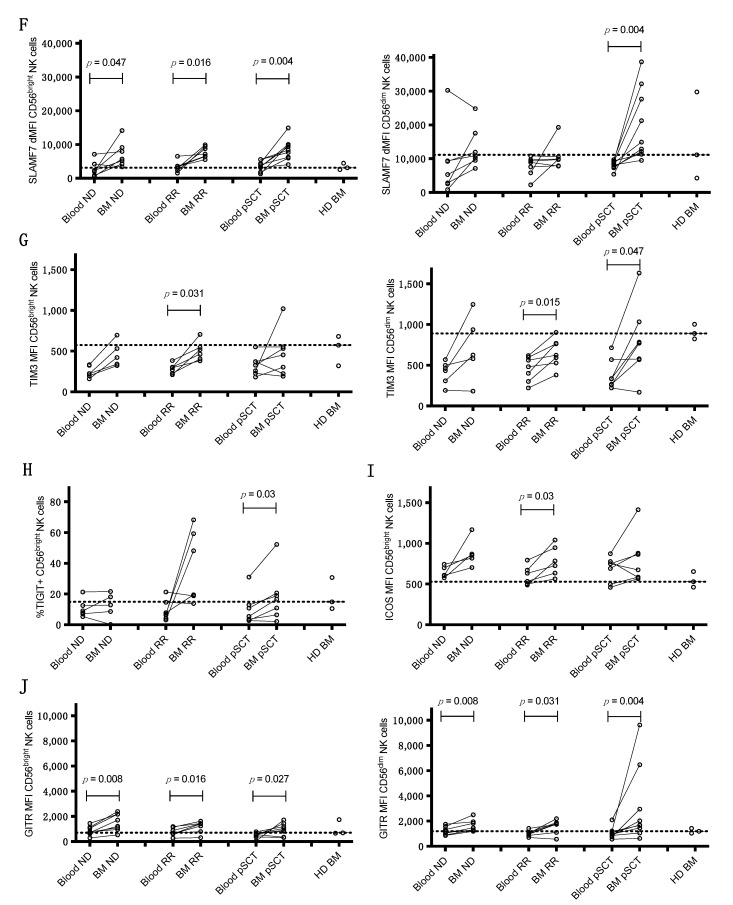
Comparisons of expression levels of various surface markers on NK cells in the blood versus bone marrow (BM) of patients in different MM disease subgroups. Values in blood and BM from each patient are connected by solid black lines. (**A**) Comparisons of % of total viable lymphocytes that are CD45^+^CD3^−^CD56^+^ NK cells in blood vs. BM. Comparison of various NK cell receptors and markers on blood vs. BM: (**B**) % CD56^dim^ NK cells that are CD57^+^, (**C**) % CD56^bright^ (left) and CD56^dim^ NK cells that are CD69^+^ (right), (**D**) % CD56^bright^ (left) and CD56^dim^ NK cells that express DNAM-1 (right), (**E**) NKG2D expression level (NKG2D dMFI) on CD56^bright^ (left) and CD56^dim^ NK cells (right), (**F**) SLAMF7 expression level (SLAMF7 dMFI) on CD56^bright^ (left) and CD56^dim^ NK cells (right), (**G**) TIM3 expression level (TIM3 MFI) on CD56^bright^ (left) and CD56^dim^ NK cells (right), (**H**) % CD56^bright^ NK cells that are TIGIT^+^, (**I**) ICOS expression level (ICOS MFI) on CD56^bright^ NK cells, (**J**) GITR expression level (GITR MFI) on CD56^bright^ (left) and CD56^dim^ NK cells (right). Dashed lines in each panel designate the median expression of each receptor on NK cells from BM of the three HDs (right), demonstrating significant shifts in expression levels in the BM of many MM patients. *p* values comparing blood to BM were calculated using Wilcoxon paired signed-rank tests.

Since BM constitutes the myeloma TME, NK cells were also analyzed in BM samples from a subset of ND, RR, and pSCT MM patients who were undergoing a concurrent BM aspiration at the time of peripheral blood sampling (10 ND, 9 RR, and 9 pSCT). Fresh BM was also obtained from three HDs, although coordinate blood samples were unfortunately not available from these HDs. Myeloma cell populations in BM were analyzed using the gating strategy outlined in Figure S3A. Direct comparisons were then made between the expression of receptors and biomarkers on NK cells in the patient BM to expression on NK cells in concurrently obtained blood samples from the same patients, as well comparisons to the expression on NK cells from the HD BM (Figure 2, and representative histograms in Figure S3B). The frequency of total NK cells was significantly lower in BM than in blood from pSCT patients, although levels were similar to the range found in BM from healthy donors (Figure 2A). The percentage of terminally mature CD57^+^ CD56^dim^ NK cells was significantly lower in BM compared to blood of ND, RR, and pSCT patients (Figure 2B), which was particularly notable in RR patients that already had reductions in these cells in the blood (Figure 1B). The CD56^bright^ NK cell subset was significantly more activated in the BM of ND and RR patient groups, as assessed by a greater fraction of cells expressing CD69 (Figure 2C), which is consistent with the higher CD69 expression on these cells in blood of these patients (Figure 1D). Notably, the expression of CD69 on BM CD56^bright^ NK cells in many of these patients was strikingly higher than levels in BM of healthy donors (Figure 2C). CD69 expression was also significantly higher on BM CD56^bright^ and CD56^dim^ NK cells from pSCT patients as compared to blood (Figure 2C), indicating an overall increased activation state of NK cells. Additionally, a large reduction in DNAM-1 expression on BM CD56^bright^ and CD56^dim^ NK cells was noted in all three patient groups (Figure 2D), which reflects reductions observed on CD56^bright^ NK cells in the blood of RR and pSCT patients and on CD56^dim^ NK cells of RR patients (Figure 1E). Importantly, the levels of DNAM-1 were markedly lower in many of these patients, as compared to expression in BM of healthy donors (Figure 2D). In contrast, the expression levels of NKG2D (Figure 2E) and SLAMF7 (Figure 2F) were significantly higher on CD56^bright^ NK cells from BM vs. blood in all three patient groups, whereas NKG2D expression on CD56^bright^ NK cells in blood was unchanged in all of these patient groups, as compared to HD blood (Figure 1F). SLAMF7 expression was also significantly higher on BM CD56^dim^ NK cells in the BM of pSCT patients (Figure 2F). In blood, the only increased expression of SLAMF7 was seen on CD56^bright^ NK cells of RR patients, as compared to HDs (Figure 1G).

The expression of two immune checkpoint receptors was also found to increase on BM-derived NK cells, as compared to those in blood. Expression levels of T-cell Ig and mucin-domain containing 3 (TIM3) were consistently higher on BM CD56^bright^ and CD56^dim^ NK cells from RR patients as compared to blood, as well as on BM CD56^dim^ NK cells from pSCT patients (Figure 2G). Of note, the levels of TIM3 on BM NK cells in patients were generally lower or equivalent to those observed in HD BM samples. The percentage of CD56^bright^ NK cells expressing T-cell immunoreceptor with Ig and ITIM domains (TIGIT) was also significantly increased in the BM of pSCT patients and in several of the RR patients (Figure 2H). In contrast, the staining of PD-1 or LAG3 on NK cells was minimal, and these were not expressed at higher levels on NK cells in patient samples, as compared to HD NK cells in this study.

In addition, increased expression of the receptors inducible T-cell costimulator (ICOS) and glucocorticoid-induced TNFR-related protein (GITR), which are potential targets for therapeutic antibodies, was noted on NK cells from the BM microenvironment of MM patients. ICOS expression levels were higher on BM CD56^bright^ NK cells from RR patients as compared to blood, and expression was higher on BM NK cells of most patients in all disease groups than on HD BM NK cells (Figure 2I). Of particular note, levels of GITR expression were significantly higher on both CD56^bright^ and CD56^dim^ NK cells in BM, as compared to the blood of all three patient groups (Figure 2J).

Importantly, the expression of most of these biomarkers was generally more uniformly expressed on NK cells from the three HD BM samples, and the levels of expression on the HD BM NK cells tended to be more similar to levels on peripheral blood NK cells of MM patients for most biomarkers (Figure 2A–J). These results further reinforce the conclusion that the myeloma TME in the BM is impacting the expression of these receptors on NK cells. 

### 2.3. Correlation of Expression of Activating Receptors on NK Cells with Ligands on BM MM Cells

The next assessment was whether the expression of receptors on NK cells in the blood or BM of ND and RR MM patients correlated with the expression of their cognate ligands on myeloma cells in the matching BM samples from the same patients. The post-SCT BM samples were excluded, since they typically had too few MM cells for informative analyses of ligand expression. The antibody panel for BM staining is presented in Table S2. Receptors and their ligands analyzed in the flow cytometry panel were: NKG2D in conjunction with UL16-binding protein (ULBP1–6) and MICA/MICB ligands, DNAM-1 and TIGIT with Nectin 2 and polio virus receptor (PVR; CD155) ligands, SLAMF7 and SLAMF7 (self-ligand), CD137 and CD137 ligand (L), PD-1 and PD-L1/2, GITR and GITR ligand (GITR-L), and ICOS and ICOS ligand (ICOS-L). Only those receptors in either CD56^dim^ or CD56^bright^ NK cells showing significantly correlated expression with a coordinate ligand on myeloma cells are shown.

**Figure 3:**
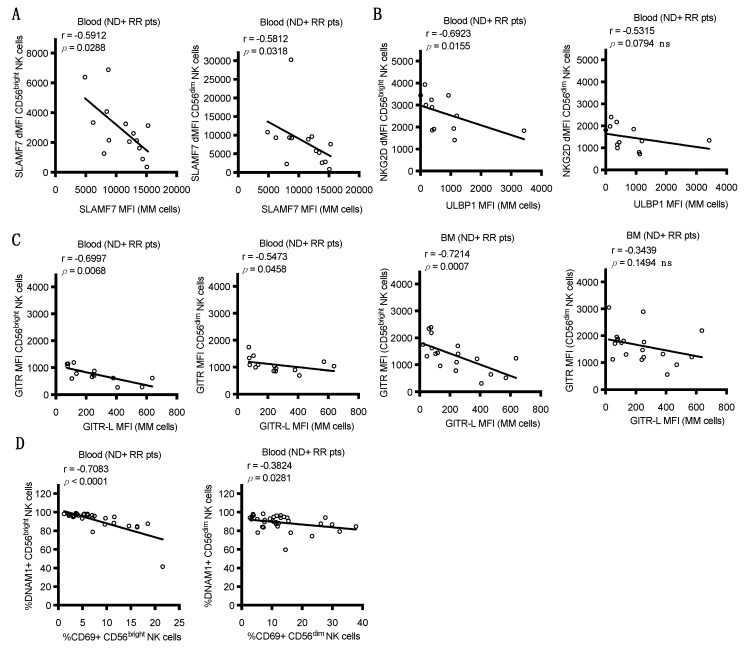
Correlations of NK cell receptor expression with ligand expression on MM cells in BM from pooled ND and RR (ND+RR) patients. Each symbol is an individual patient. Statistics were calculated with Spearman’s rank correlation coefficient, lines are linear least squares fit for visual purposes only. Patients with corresponding blood and BM samples from ND+RR patients were examined for correlations between expression levels of the indicated receptors (y-axes) in blood or BM (as indicated) and expression of their corresponding ligands on myeloma cells in the BM (x-axes). (**A**) Correlations between dMFI of SLAMF7 expression on blood CD56^bright^ (left) and CD56^dim^ NK cells (right) and on myeloma (MM) cells in BM. (**B**) Comparisons of dMFI of NKG2D expression on blood CD56^bright^ (left) and CD56^dim^ NK cells (right) and the NKG2D ligand, ULBP1, on MM cells in BM. (**C**) Correlations between MFI expression levels of GITR on NK cell populations in blood (left panels) or in BM (right panels) with expression of GITR-L on MM cells in the BM. (**D**) Correlation between % CD56^bright^ (left) or CD56^dim^ (right) NK cells in blood that are DNAM-1^+^ and their % expressing the CD69 activation marker.

A significant negative correlation was noted between the expression of SLAMF7 on myeloma cells of the BM and SLAMF7 expression on either CD56^dim^ or CD56^bright^ NK cells in the peripheral blood of ND and RR patients (Figure 3A). In addition, a significant negative correlation was found between the expression of ULBP1 ligand on myeloma cells in BM and the expression of NKG2D on blood CD56^bright^ NK cells of ND and RR patients, which was also trending on CD56^dim^ NK cells (Figure 3B). In addition, when focusing only on ND patients, the expression of the NKG2D ligand, ULBP3, on BM myeloma cells negatively correlated with the expression level of NKG2D on both CD56^bright^ and CD56^dim^ NK cells in BM (Figure S4). Furthermore, a significant negative correlation was observed between GITR-L levels on myeloma cells in the BM and the expression of GITR on CD56^bright^ NK cells in both blood and BM, whereas a negative correlation was less apparent on CD56^dim^ NK cells in blood and not significant in the BM (Figure 3C). As previously shown for NKG2D [28], these results suggest that the expression of SLAMF7, NKG2D, and GITR may be downregulated when they encounter high levels of their ligands on myeloma cells or perhaps soluble ligands shed from tumors (not measured in this study). In support of this hypothesis, a negative correlation was observed between the expression of DNAM-1 and the expression of CD69 on both CD56^bright^ and CD56^dim^ NK cells in blood (Figure 3D), suggesting that ligand-induced engagement is downregulating DNAM-1 and activating NK cells in the patients. In contrast, the expression levels of DNAM-1, TIGIT, CD137, PD-1, or ICOS did not significantly correlate with the expression of their corresponding ligands.

### 2.4. Correlations of NK Cell Biomarker Expression with Progression-Free Survival of MM Patients

The next analysis asked whether the expression levels of the NK cell surface markers found to be altered in the blood of MM patients correlated with length of progression-free survival. For this analysis, the ND, RR, and pSCT MM patients were pooled and these patients were divided into tertiles based on expression level of the specific surface biomarker on NK cells in PBMCs to generate Kaplan–Meier plots. Statistical analysis was performed with Mantel–Cox (M–C) comparison of the tertiles and Cox proportional hazards (CPH) regression analysis of each total dataset.

This tertile-based analysis of surface biomarkers on NK cells in the pooled ND+RR+pSCT patients revealed a shorter time to progression (worse outcome) in patients exhibiting a lower percentage of DNAM-1 expression on CD56^dim^ and CD56^bright^ NK cells (Figure S5A), a higher expression of CD69 on immature CD56^bright^ NK cells (Figure S5B), and a lower percentage of terminally mature CD57^+^CD56^dim^ NK cells in blood (Figure S5C). However, these correlations may be driven by RR patients, who were more prevalent in the high-risk tertiles, and are more likely to exhibit these progression-associated NK cell biomarker phenotypes (Figure 1B,D,E). Analysis of these parameters in the RR group alone did not demonstrate statistically significant correlations to progression-free survival (PFS).

**Figure 4:**
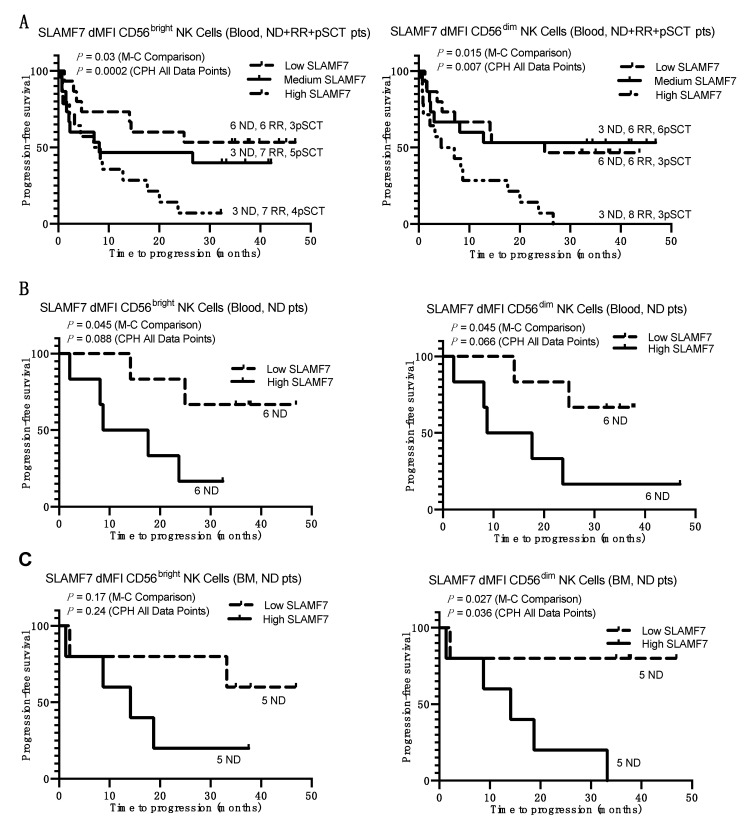
Kaplan–Meier survival plots showing time to progression as a function of SLAMF7 expression on NK cells in blood and BM. (**A**) Plots for the combined ND+ RR+ pSCT MM patients divided into tertiles based on their dMFI values for SLAMF7 expression on CD56^bright^ (left) and CD56^dim^ NK cells (right) in blood. Numbers of patients in each tertile that are ND, RR, or pSCT are indicated adjacent to each plotted line. (**B**,**C**) Plots for only ND MM patients divided into halves above and below the median of dMFI values for SLAMF7 expression on their CD56^bright^ (left) and CD56^dim^ NK cells (right) in peripheral blood (**B**) and BM (**C**). Line designations for high-, medium-, and low-expression tertiles and halves are indicated in the individual panels. *p* values are listed for a Cox proportional hazards (CPH) test performed on all data points and Mantel–Cox (M–C) statistical analysis of each patient group divided into tertiles or halves.

Using the tertile-based analysis of pooled ND+RR+pSCT patients, statistically significant correlations were also found between levels of SLAMF7 expression on both CD56^dim^ and CD56^bright^ NK cells in blood and PFS (Figure 4A), with the shortest PFS in the tertile with the highest SLAMF7 expression. Importantly, the numbers of RR patients were well distributed between these tertiles, demonstrating that these correlations were not driven by a phenotype that is concentrated in RR patients, who are expected to have shorter PFS. We further focused on individual patient groups, which were analyzed for PFS of the above and below median halves of SLAMF7 expression levels on NK subsets in blood and BM. This analysis was performed by dividing the patients into halves, since the individual ND, RR, and pSCT groups were smaller than when combined. As shown in Figure 4B, ND patients with higher levels of SLAMF7 on either CD56^dim^ or CD56^bright^ NK cells in blood had significantly worse PFS, with a similar, but non-significant trend observed in pSCT patients (Figure S6A). This association was absent in the RR patients (Figure S6B). Furthermore, ND patients with a high expression of SLAMF7 on CD56^dim^ NK cells in BM also had significantly worse PFS (Figure 4C). Taken together, the data suggest that MM patients with early high levels of SLAMF7 expression on NK cells in blood or BM have shorter PFS regardless of subsequent therapy, and this correlation was particularly evident in ND patients.

## 3. Discussion

Here, we performed an observational study of NK cell status in an unselected cohort of MM patients at different stages of disease, namely SMM, ND, RR, and pSCT. Our goal was to establish NK cell phenotype and function in these patients and potentially identify prognostic biomarkers or new therapeutic targets. We acknowledge the weaknesses of the study, namely, small cohorts of patients in each stage of MM and variability of clinical characteristics between these groups (e.g., higher proportion of extramedullary complications in the RR group compared to ND), but several intriguing differences in NK cell phenotype were established. Of course, these differences need to be validated in additional patient cohorts. As summarized in Table 2, when the expression of biomarkers on NK cells from HD was compared to each of the MM subtypes, most of the differences were concentrated in RR and pSCT patients, where the NK cells tend to be less mature (increased immature CD56^bright^ cells and lower expression of FcγRIIIa (CD16) and KIR2DL1/S1 on CD56^dim^ NK cells; Figure 1). The evidence of fewer mature cells in RR and pSCT patients likely reflects ongoing immune reconstitution after chemotherapy and SCT. Expression of the CD69 activation marker was also higher on CD56^bright^ NK cells in ND patients and on both CD56^bright^ and CD56^dim^ NK cells in RR patients compared to HDs (Figure 1D). CD69 expression was further increased on CD56^bright^ NK cells in BM, as compared to blood, in ND, RR, and pSCT patients (Figure 2C).

The expression of DNAM-1 (CD226) was reduced on peripheral NK cells in RR and pSCT MM patients compared to HDs (Figure 1E), and the expression of DNAM-1 on NK cells was even lower in the BM TME in ND, RR, and pSCT MM patients, as compared to peripheral blood (Figure 2D). Interestingly, the reduced expression of DNAM-1 correlated with increased expression of CD69 on NK cells in the blood of ND and RR patients (Figure 3D). This is consistent with reduced DNAM-1 and increased CD69 expression on NK cells exposed in vitro to tumor target cells expressing DNAM-1 ligands [29]. Taken together, these observations suggest that NK cells are becoming activated upon DNAM-1 engagement with ligands on BM myeloma cells, which results in the subsequent downregulation or shedding of the receptor from the NK cell surface. Nonetheless, we did not observe a direct correlation between the expression of DNAM-1 on NK cells in blood or BM and the expression of ligands (PVR and nectin2) on myeloma cells in BM. Consistent with our results, Vulpis et al. recently reported significantly reduced expression of DNAM-1 on CD56^bright^ (and a small subset of CD56^dim^CD16^low^) NK cells in RR MM patients [30], and El-Sherbiny et al. found reduced expression on CD56^dim^ NK cells from patients with active MM, but not from patients that have had a complete response to therapy [23]. The reduced expression of DNAM-1 has been observed in a variety of other cancers, including a recent report by Guillamon et al., showing lower overall survival in patients with solid tumors that exhibit a low expression of DNAM-1 on NK cells [31]. While we observed worse PFS in combined ND, RR, and pSCT patients expressing the lowest levels of DNAM-1 (Figure S5A), this was driven by the low expression of DNAM-1 and a higher risk of relapse in the RR patient subgroup.

Overall, significant reductions in the expression of NKG2D were found on CD56^dim^ NK cells in the blood of RR and pSCT patients, as compared to HDs (Figure 1F), but levels were significantly higher in the BM TME on CD56^bright^ cells (and on CD56^dim^ in some RR patients), as compared to blood, in ND, RR, and pSCT patients (Figure 2E). On the other hand, we found that high levels of ULBP1 ligand on BM myeloma cells correlated with reduced expression of NKG2D on CD56^bright^ NK cells in peripheral blood (Figure 3B), suggesting ligand-induced downregulation. A similar negative correlation with ULBP3 expression on myeloma and NKG2D on BM NK cells was only evident in the BM of ND patients (Figure S4), which is consistent with evidence from Carbone et al. that NKG2D ligands are most elevated on myeloma cells in the BM of early-stage patients [21]. We conclude that ULBP3 may either be playing a more important role in promoting NK cell attack in early-stage ND patients or that ULBP3 may be shed from the surface of myeloma cells in later-stage disease. Others have previously reported a decreased expression of NKG2D on NK cells in MM patients [32], and a loss of NKG2D expression is associated with NK cell exhaustion in the TME [33]. This loss of NKG2D expression may be due to the encounter of NK cells with ligands expressed on MM tumor cells or the shedding of ligands into the plasma of MM patients [28,34]. Regardless of the mechanism, the reduction of NKG2D expression would result in limiting NK cell recognition of ligand-bearing myeloma cells.

NKG2D and DNAM-1 have previously been found to play significant roles in the NK cell-mediated attack of myeloma cells, and their loss could contribute to NK cell dysfunction. Multiple groups have reported the suppression of the in vitro NK cell-mediated cytotoxicity of several myeloma lines by antibodies blocking either DNAM-1 or NKG2D, and this effect was dependent upon ligand expression [21,23]. Additionally, in an MM mouse model, the knockout of DNAM-1 severely compromised the anti-myeloma immune response and diminished the anti-myeloma efficacy of cyclophosphamide and bortezomib [25]. The exposure of myeloma cells to doxorubicin, melphalan, or bortezomib upregulates DNAM-1 and NKG2D ligands through initiating a DNA damage response, an ATM-dependent activation of p53, and inducing senescence, which may contribute to the therapeutic mechanism of these agents [9,35,36]. Lenalidomide and pomalidomide, which are also commonly used to treat MM, can also upregulate DNAM-1 and NKG2D ligands through the downregulation of the transcription factors Ikaros (IKZF1) and Aiolos (IKZF3) that negatively regulate their transcription [37].

The expression of additional activating receptors was altered in our MM patient cohorts. NCRs play important roles in anti-tumor responses by NK cells [5], and RR patients had an increased expression of NKp30 and NKp46, but a reduced expression of NKp44 in the blood of RR patients, as compared to HDs (Figure 1I). Furthermore, CD16 (FcγRIIIa) expression was reduced on CD56^dim^ NK cells in the blood of in SMM, RR, and pSCT patients (Figure 1C). Despite this reduction, the degranulation of NK cells was not reduced under ADCC-inducing conditions with elotuzumab (Figure S2), suggesting NK cells in MM patients retain capacity for ADCC toward myeloma when treated with elotuzumab. Fauriat et al. also previously found a reduced expression (MFI) of CD16 on NK cells in MM patients, although levels of NKp30, NKp44, or NKp46 did not differ from HDs [24]. Importantly, El-Sherbiny et al. found that the blockade of NKp46 inhibited the in vitro killing of all of the myeloma lines tested [23], suggesting that increased NKp46 expression in RR patients may be beneficial, although we did not observe a correlation of NKp46 with PFS. Carbone et al. showed reduced in vitro cytotoxicity of myeloma target cells by NK cells upon blocking all three NCRs together with a pool of antibodies, and the further addition of NKG2D antibody was even more effective [21]. We did not, however, observe any altered degranulation response by NK cells from any of the MM patient subgroups under natural cytotoxicity conditions toward MM.1R target cells, as compared to HDs (Figure S2). In contrast, two studies have found progressively reduced cytotoxicity of K562 target cells with NK cells from MM patients with increasing disease stage [38,39]. The reduced cytotoxicity in those studies may be unique to the K562 target cell, and we did not directly measure target cytotoxicity.

The expression of the co-stimulatory receptors, GITR and ICOS, was also altered in MM patients. Levels of GITR were increased on CD56^bright^ and CD56^dim^ NK cells in BM as compared to blood in ND, RR, and pSCT patients (Figure 2J), and ICOS expression was higher on the BM CD56^bright^ NK cells of RR patients (Figure 2I). Furthermore, a negative correlation was noted between GITR ligand expression on BM myeloma cells and GITR expression on CD56^bright^ NK cells in the blood and BM of ND and RR patients (Figure 3C). The disruption of GITR–GITR-L interactions enhanced NK cell activation in pre-clinical acute myeloid leukemia and chronic lymphocytic leukemia models [40,41]. The ligation of ICOS can enhance the activation of NK cells [42] and agonist antibodies against ICOS have entered clinical trials [43]. These data provide a preliminary rationale for exploring targeting these pathways in MM.

We also examined the expression of several immune checkpoint receptors on NK cells in our MM patient samples. A higher expression of TIGIT and TIM3 were found on NK cells in the BM of MM patients, as compared to levels in the blood (Figure 2G,H), suggesting upregulation in the TME. Both TIGIT and TIM3 have been shown to potentially suppress NK cell function, suggesting that therapeutic antibodies targeting these receptors may enhance NK cell function in at least some MM patients [44]. We note, however, that the measured levels were only substantially higher in a few patients as compared to levels on NK cells from the BM of HDs. Recent studies found that TIGIT-blocking antibodies or TIGIT knockout potentiated anti-myeloma responses in mouse models, although this was primarily attributed to TIGIT expression suppressing the activation capacity of CD8^+^ T cells [45,46]. Trials of anti-TIGIT antibodies in MM patients, alone and in combination with daratumumab or elotuzumab, are underway (ClinicalTrials.gov Identifiers: NCT04045028, NCT04150965, www.clinicaltrials.gov). Importantly, while Benson et al. previously reported an increased expression of PD-1 on NK cells from MM patients [17], we did not detect appreciable expression of PD-1 on NK cells in our samples using the same antibody clone (MIH4). Furthermore, the PD-1-blocking antibody (CT-011, pidilizumab) used to enhance anti-myeloma cytotoxicity responses by NK cells in that report was subsequently found to primarily target the Notch2 ligand, Delta-like 1 (DLL1) [47,48]. Overall, as compared to T cells, we find that PD-1 expression levels are almost negligible on NK cells in a variety of cancers, even when stained with nivolumab or pembrolizumab. Therefore, we conclude that PD-1 is not appreciably upregulated on NK cells in MM patients and preclinical support for the use of PD-1 blockade to enhance anti-tumor responses by NK cells in MM patients is unclear. To date, PD-1-blocking therapies have shown limited efficacy in treating MM, although potential combination therapies may still prove beneficial, such as with the blockade of TIGIT, TIM3, and/or LAG3 [49,50].

One of the most striking impacts of MM that we observed on NK cells was the increased expression of SLAMF7 and a significant correlation of SLAMF7 expression with PFS. SLAMF7 engages with itself as a ligand and is highly expressed on both NK cells and MM cells [10]. We have previously shown that coordinate SLAMF7 expression on both NK cells and myeloma target cells significantly enhances natural cytotoxicity responses, since SLAMF7 is a co-stimulatory receptor in NK cells [51]. Here, we found substantially higher SLAMF7 levels on NK cells in BM, as compared to peripheral blood, in nearly all ND, RR, and pSCT patients, and the SLAMF7 expression levels on CD56^bright^ NK cells in the BM of most MM patients was higher than in the BM of three healthy controls (Figure 2F). Furthermore, patients with high levels of SLAMF7 expression on both CD56^bright^ and CD56^dim^ NK cells in the blood had worse clinical outcomes, as measured by shorter PFS (Figure 4A). This impact was particularly evident in ND patients, who exhibited a shorter time to progression if SLAMF7 was expressed at high levels on CD56^bright^ and CD56^dim^ NK cells in blood, as well as CD56^dim^ NK cells in BM (Figure 4B,C). These results implicate a high expression of SLAMF7 on NK cells as a potential biomarker of poor outcome in previously untreated ND patients that subsequently received standard of care therapy, and merit further study in larger datasets to try to validate this finding. Importantly, we also found that patients with high SLAMF7 expression on NK cells had lower SLAMF7 expression on MM tumors (Figure 3A). This observation suggests that the SLAMF7–SLAMF7 interactions between NK cells and MM tumors may be important for mediating more efficient tumor control. Since these patients received standard of care therapy, it is not known if SLAMF7 expression on NK cells would retain the same prognostic value for patients treated with the SLAMF7-targeting antibody elotuzumab. Therefore, it may be worthwhile to explore if ND patients with high SLAMF7 expression on NK cells are more responsive to treatment with elotuzumab, which stimulates the antibody-dependent killing of myeloma target cells by NK cells and monocytes and promotes the SLAMF7–SLAMF7-dependent co-stimulation of NK cells [10,51,52].

## 4. Materials and Methods 

### 4.1. Patient Selection

Patients were recruited by written informed consent following HIPAA-compliant procedures approved by the Institutional Review Boards (IRB) of the University of Pennsylvania (Penn) and Fox Chase Cancer Center (FCCC), in accordance with the Declaration of Helsinki. All protocols underwent yearly IRB renewal during the course of the project. Recruitment was based on clinical records of myeloma patients undergoing evaluation or treatment at Penn and diagnosed within four disease stages: smoldering (SMM), newly diagnosed active MM prior to starting therapy (ND), relapsed/refractory just prior to starting a new regimen (RR), and about 100 days post-high-dose melphalan and autologous stem cell transplant (pSCT). Patient characteristics are summarized in Table 1. Blood samples were also acquired from healthy donors after HIPAA-compliant consent approved by the FCCC IRB. Bone marrow samples were obtained from three healthy donors after HIPAA-compliant consent approved by the Penn IRB. Patient samples were collected between November 2013 and May 2016. The cut-off for collection of the follow-up clinical data was 11/1/18. The retrospective analysis of clinical data was approved by the ethical committees of both institutions.

### 4.2. Assessment of Response Criteria

The definitions of smoldering versus active myeloma, evaluation of response, and assessment of disease progression were conducted in accordance with updated International Myeloma Working Group criteria [53]. Progression-free survival (PFS) was defined as the time from sample collection to disease progression or death, whichever occurred first. Patients who were alive and progression free at last contact were censored at that time.

### 4.3. Phenotyping of Blood and Bone Marrow Samples by Flow Cytometry

Whole blood (20 mL) and bone marrow (BM; 5–10 mL) samples from MM patients and HD volunteers were collected by venipuncture or marrow aspiration, respectively, into heparinized tubes (#366480, BD Biosciences Inc., San Jose, CA, USA) and kept at room temperature before processing and analysis within 6 h. PBMCs were purified from blood samples using Lymphoprep (Axis-Shield POC AS, Oslo, Norway) and gradient centrifugation, as previously described [54,55]. BM aspirates were treated for 5 min on ice with red cell lysis buffer (0.155 M NH_4_Cl, 1 mM Na_2_EDTA, 0.01 M KHCO_3_), washed 1× with red cell lysis buffer, and passed through a Falcon 40 µm strainer (#352340, Corning, Corning, NY, USA) to obtain leukocytes. PBMCs and BM leukocytes were washed 2× with Hanks’ Balanced Salt solution (HBSS) and resuspended in complete RPMI-1640 medium (supplemented with 10% fetal bovine serum (FBS), 100 µg/mL penicillin/streptomycin, 2 mM L-glutamine, 10 mM HEPES, 1 mM sodium pyruvate, and 50 µM 2-mercaptoethanol) at 10 million cells/mL. Individual one million cell aliquots were stained with panels of up to 10 fluorescently conjugated antibodies (as detailed in Tables S1 and S2) for 20 min on ice and washed twice with ice-cold wash buffer (HBSS + 10% FBS + 0.1% Na Azide), with the final wash buffer containing propidium iodide (PI; 100 ng/mL). SLAMF7 was stained with elotuzumab conjugated with Allophycocyanin (APC; EZ-Link NHS-Biotin, #20217, Thermo Fisher, Walltham, MA, USA). For antibodies requiring secondary antibodies, staining was performed for an additional 20 min on ice, followed by two washes.

### 4.4. NK Cell Degranulation Assay

NK cell degranulation was assessed as previously described [54]. Aliquots of one million freshly isolated PBMCs were incubated for 2 h at 37 °C in 200 µL complete RPMI-1640 medium under four stimulation conditions: leukocytes alone, +1 µg/mL elotuzumab, + MM.1R target cells (one million cells), or + both MM.1R and elotuzumab. Samples were centrifuged at 150 relative centrifugal force (RCF) for 3 min before incubation. Anti-CD107a-PE (H4A3, BD Biosciences Inc., San Jose, CA, USA), anti-CD45-PerCP-Cy5.5 (2D1, eBioscience, San Diego, CA, USA), anti-CD3-APC-H7 (SK7, BD Biosciences Inc., San Jose, CA, USA), and anti-CD56-APC (NCAM 16.2, Biolegend, San Diego, CA, USA) antibodies were added in the last 30 min of culture. Cells were centrifuged and rinsed twice with ice-cold wash buffer, with PI added in the second wash. NK cell degranulation was measured as a percentage of CD107a^+^ cells among total PI^−^CD45^+^CD3^−^CD56^dim^ NK cells.

### 4.5. Flow Cytometry and Data Analysis

Flow cytometry was performed on a BD Aria II cytometer (BD Bioscience, San Jose, CA, USA) fitted with four diode lasers with excitation wavelengths of 633, 488, 405, and 355 nm. The cytometer was located in the FCCC Clinical Pathology Flow Cytometry Laboratory, which is CLIA (The Clinical Laboratory Improvement Amendments of 1988)- and CAP (College of American Pathologists)-certified, and calibrated daily with fluorescent BD CompBeads. Between 100,000 to 400,000 events were acquired from each sample at 500–2500 events/second using 70 psi (pounds per square inch) pressure. Compensation and photomultiplier tube (PMT) voltage settings were consistent for the acquisition of all samples, as optimized at the beginning of the project based upon the analysis of unstained, single-stained, and multi-stained PBMC samples. Data were collected with BD FACS Diva software version 6 and analyzed using Flow Jo software (v9.2 or later; BD Biosciences Inc.), Microsoft Excel (v12; Redmond, WA, USA), GraphPad Prism (v5.0d or later; GraphPad Software, Inc., San Diego, CA, USA), and R (The R Foundation; www.r-project.org). All raw data were processed manually to ensure data consistency of the gating strategy, with examples provided in Figure S1. Positive staining was determined either by selecting the expressing subset of a clearly bimodal population, by comparison to staining in a comparable tube stained with an isotype control conjugated with the same fluorophore (NK cell panel), or by comparison to cellular subsets in the same tube that do not express the marker in question (MM ligands panel). Biomarker parameters were quantified as a percentage of expressing cells or mean fluorescence intensity (MFI) of staining, and these were compared between the five groups (healthy controls, SMM, ND, RR, and pSCT) or between blood and BM samples to identify statistically significant differences. Some parameters (NKG2D, SLAMF7, NKp30, and NKp46) are presented as delta MFI (dMFI), which is the MFI of staining with the target antibody minus the MFI of staining with an isotype control antibody. The gating of myeloma cells in the BM samples was guided by a hematopathologist (E.D.) A representative example of myeloma cell gating is shown in Figure S2.

### 4.6. Statistical Analysis

Data are presented as mean fluorescent intensity (MFI) or percentages of positive populations. Statistical significance was evaluated using a nonparametric Mann–Whitney test to compare MM vs. HD samples and a nonparametric paired Wilcoxon signed-rank test to compare parameters in blood vs. bone marrow from the same patients. The Spearman test was used for testing the statistical significance of correlations between parameters. PFS was analyzed using the Kaplan–Meier method. Statistical significance was determined using a Cox proportional hazards regression analysis of each total dataset and Mantel–Cox comparisons of patients divided into tertiles or halves. Statistical analysis and plots were generated in GraphPad Prism (v5.0d or later; GraphPad Software, Inc., San Diego, CA, USA). All tests were two-sided. Statistical significance was considered as *p* < 0.05.

## 5. Conclusions

In conclusion, this prospective study of NK cells in MM patients has revealed several new insights of potential clinical relevance. Consistent with several previous studies of NK cells, in comparison to HDs, a lower expression was found for several activating receptors, such as DNAM-1, NKG2D, CD16, and NKp44, which may contribute to attenuated anti-tumor response, but paradoxical increased expression was observed for NKp30, NKp46, and LFA-1 (CD11a). These changes in activating receptors were mostly found in RR and pSCT patients. In addition, increased expression of several potential therapeutic target receptors was found on BM NK cells, namely TIM3, TIGIT, ICOS, and GITR. An important feature of this study is the identification of several prominent NK cell changes in the BM TME that are also evident in the blood, which makes their detection relatively accessible. In particular, we observed increased expression of CD69 and SLAMF7 and decreased CD57 and DNAM-1 on blood NK cells, especially in RR patients, and these changes were even more pronounced in BM. Notably, comparisons of biomarker expression on NK cells and patient outcomes revealed that a high expression of SLAMF7 on NK cells correlated with shorter PFS, which was particularly evident in the blood and BM of ND patients.

## Figures and Tables

**Table 1:** Characteristics of patients and healthy donors.

Patient Characteristic	HD (*n* = 19)	SMM (*n* = 7)	ND (*n* = 17)	RR (*n* = 23)	pSCT (*n* = 14)
Age, median (range)	63 (40–91)	69 (47–78)	65 (40–82)	62 (36–86)	61 (41–74)
MM isotype, % IgG/IgA/Light chain only	-	86/14/0	53/35/12	52/17/26	64/0/36
High risk cytogenetics *	-	29%	56%	67%	56%
Extramedullary disease, % Present/Absent/Unknown	-	0/100/0	18/53/29	39/48/13	14/79/7
Prior lines **, median (range)	-	-	-	5 (1–10)	1 (1–2)
Prior Lenalidomide, %	-	-	-	100%	57%
Prior Thalidomide, %	-	-	-	35%	0%
Prior Pomalidomide, %	-	-	-	61%	0%
Prior Bortezomib, %	-	-	-	96%	100%
Prior Carfilzomib, %	-	-	-	74%	0%
Prior daratumumab, %	-	-	-	4%	0%
Prior cyclophosphamide, %	-	-	-	83%	57%
Prior Dexamethasone, %	-	-	-	100%	100%
Prior SCT, %	-	-	-	74%	100%
Absolute Lymphocyte Count (×10^3^/µL), median (range)	-	1.47 (0.49–3.77)	1.84 (0.70–3.60)	0.6 (0.2–2.6)	0.94 (0.34–3.44)

* Includes t(4;14), t(14;16), t(14;20), gain 1q, loss 1p, del 17p, complex karyotype. ** Lines of therapy determined as per International Myeloma Working Group criteria. HD = healthy donor; ND = newly diagnosed; pSCT = post-autologous stem cell transplant; RR = relapsed/refractory; SMM = smoldering multiple myeloma.

**Table 2:** Summary of significantly increased (↑) or decreased (↓) expression of biomarkers on the indicated NK cell subset in MM patient subgroups compared to healthy controls and in bone marrow compared to the blood of patients.

Parameter	CD56^bright/dim^	SMM	ND	RR	pSCT	Difference in BM Compared to Blood
Markers of Maturation and Activation						
% NK of lymphocytes	total					↓ pSCT
% NK subset	dim			↓	↓	
CD57	dim			↓	↓	↓ ND, RR, pSCT
KIR2DL1/S1	dim			↓	↓	
CD69	bright		↑	↑		↑ ND, RR, pSCT
	dim			↑		↑ pSCT
Activating Receptors						
CD16	dim	↓		↓	↓	
DNAM-1	bright			↓	↓	↓ ND, RR, pSCT
	dim			↓		↓ ND, RR, pSCT
NKG2D	bright					↑ ND, RR, pSCT
	dim			↓	↓	
SLAMF7	bright			↑		↑ ND, RR, pSCT
	dim					↑ pSCT
CD11a	bright			↑	↑	
	dim				↑	
NKp30	dim			↑		
NKp46	bright			↑		
	dim			↑	↑	
NKp44	bright			↓		
Therapeutic Targets						
TIM3	bright					↑ RR
	dim					↑ RR, pSCT
TIGIT	bright					↑ pSCT
ICOS	bright					↑ RR
GITR	bright					↑ ND, RR, pSCT
	dim					↑ ND, RR, pSCT

## Data Availability

The data presented in this study are available upon reasonable request from the corresponding authors. The data are not publicly available due to privacy concerns and the large volume of data.

## Supplementary Materials

The following are available online at https://www.mdpi.com/2072-6694/13/2/226/s1, Figure S1: Representative gating and staining of NK cells, Figure S2: Effects of elotuzumab on NK cell degranulation, Figure S3: BM gating, Figure S4: Correlation of NKG2D expression on BM NK cells with ULBP3 ligand expression on MM cells in BM of ND patients, Figure S5: Kaplan–Meier survival plots showing time to progression based on the expression of various NK cell markers for the combined ND, RR, and pSCT MM patients divided into tertiles, Figure S6: Kaplan–Meier survival plots showing time to progression as a function of SLAMF7 expression on blood NK cells in pSCT and RR MM patients; Table S1: NK cell antibody staining panel for flow cytometry, Table S2: Ligands on MM cell antibody staining panel for BM samples assessed by flow cytometry.

## References

[1] Campbell K.S., Hasegawa J. (2013). Natural killer cell biology: An update and future directions. J. Allergy Clin. Immunol..

[2] Cooper M.A., Fehniger T.A., Caligiuri M.A. (2001). The biology of human natural killer-cell subsets. Trends Immunol..

[3] Cooper M.A., Fehniger T.A., Turner S.C., Chen K.S., Ghaheri B.A., Ghayur T., Carson W.E., Caligiuri M.A. (2001). Human natural killer cells: A unique innate immunoregulatory role for the CD56^bright^ subset. Blood.

[4] Caligiuri M.A. (2008). Human natural killer cells. Blood.

[5] Pazina T., Shemesh A., Brusilovsky M., Porgador A., Campbell K.S. (2017). Regulation of the functions of natural cytotoxicity receptors by interactions with diverse ligands and alterations in splice variant expression. Front. Immunol..

[6] Siegel R.L., Miller K.D., Jemal A. (2020). Cancer statistics, 2020. CA Cancer J. Clin..

[7] Hayashi T., Hideshima T., Akiyama M., Podar K., Yasui H., Raje N., Kumar S., Chauhan D., Treon S.P., Richardson P. (2005). Molecular mechanisms whereby immunomodulatory drugs activate natural killer cells: Clinical application. Br. J. Haematol..

[8] Shi J., Tricot G.J., Garg T.K., Malaviarachchi P.A., Szmania S.M., Kellum R.E., Storrie B., Mulder A., Shaughnessy J.D., Barlogie B. (2008). Bortezomib down-regulates the cell-surface expression of HLA class I and enhances natural killer cell-mediated lysis of myeloma. Blood.

[9] Soriani A., Zingoni A., Cerboni C., Iannitto M.L., Ricciardi M.R., Di Gialleonardo V., Cippitelli M., Fionda C., Petrucci M.T., Guarini A. (2009). ATM-ATR-dependent up-regulation of DNAM-1 and NKG2D ligands on multiple myeloma cells by therapeutic agents results in enhanced NK-cell susceptibility and is associated with a senescent phenotype. Blood.

[10] Campbell K.S., Cohen A.D., Pazina T. (2018). Mechanisms of NK cell activation and clinical activity of the therapeutic SLAMF7 antibody, elotuzumab in multiple myeloma. Front. Immunol..

[11] Moreno L., Perez C., Zabaleta A., Manrique I., Alignani D., Ajona D., Blanco L., Lasa M., Maiso P., Rodriguez I. (2019). The mechanism of action of the Anti-CD38 Monoclonal antibody isatuximab in multiple myeloma. Clin. Cancer Res..

[12] Nijhof I.S., van Bueren J.J.L., van Kessel B., Andre P., Morel Y., Lokhorst H.M., van de Donk N.W., Parren P.W., Mutis T. (2015). Daratumumab-mediated lysis of primary multiple myeloma cells is enhanced in combination with the human anti-KIR antibody IPH2102 and lenalidomide. Haematologica.

[13] Tai Y.T., Mayes P.A., Acharya C., Zhong M.Y., Cea M., Cagnetta A., Craigen J., Yates J., Gliddon L., Fieles W. (2014). Novel anti-B-cell maturation antigen antibody-drug conjugate (GSK2857916) selectively induces killing of multiple myeloma. Blood.

[14] Gabriel I.H., Sergeant R., Szydlo R., Apperley J.F., DeLavallade H., Alsuliman A., Khoder A., Marin D., Kanfer E., Cooper N. (2010). Interaction between KIR3DS1 and HLA-Bw4 predicts for progression-free survival after autologous stem cell transplantation in patients with multiple myeloma. Blood.

[15] Kroger N., Zabelina T., Berger J., Duske H., Klyuchnikov E., Binder T., Stubig T., Hilde-brandt Y., Atanackovic D., Alchalby H. (2011). Donor KIR haplotype B improves progression-free and overall survival after allogeneic hematopoietic stem cell transplantation for multiple myeloma. Leukemia.

[16] Alici E., Sutlu T., Bjorkstrand B., Gilljam M., Stellan B., Nahi H., Quezada H.C., Gahrton G., Ljunggren H.G., Dilber M.S. (2008). Autologous antitumor activity by NK cells expanded from myeloma patients using GMP-compliant components. Blood.

[17] Benson D.M., Bakan C.E., Mishra A., Hofmeister C.C., Efebera Y., Becknell B., Baiocchi R.A., Zhang J., Yu J., Smith M.K. (2010). The PD-1/PD-L1 axis modulates the natural killer cell versus multiple myeloma effect: A therapeutic target for CT-011, a novel monoclonal anti-PD-1 antibody. Blood.

[18] Chu J., Deng Y., Benson D.M., He S., Hughes T., Zhang J., Peng Y., Mao H., Yi L., Ghoshal K. (2014). CS1-specific chimeric antigen receptor (CAR)-engineered natural killer cells enhance in vitro and in vivo antitumor activity against human multiple myeloma. Leukemia.

[19] Shah N., Li L., McCarty J., Kaur I., Yvon E., Shaim H., Muftuoglu M., Liu E., Orlowski R.Z., Cooper L. (2017). Phase I study of cord blood-derived natural killer cells combined with autologous stem cell transplantation in multiple myeloma. Br. J. Haematol..

[20] Szmania S., Lapteva N., Garg T., Greenway A., Lingo J., Nair B., Stone K., Woods E., Khan J., Stivers J. (2015). Ex vivo-expanded natural killer cells demonstrate robust proliferation in vivo in high-risk relapsed multiple myeloma patients. J. Immunother..

[21] Carbone E., Neri P., Mesuraca M., Fulciniti M.T., Otsuki T., Pende D., Groh V., Spies T., Pollio G., Cosman D. (2005). HLA class I, NKG2D, and natural cytotoxicity receptors regulate multiple myeloma cell recognition by natural killer cells. Blood.

[22] Casneuf T., Xu X.S., Adams H.C., Axel A.E., Chiu C., Khan I., Ahmadi T., Yan X., Lonial S., Plesner T. (2017). Effects of daratumumab on natural killer cells and impact on clinical outcomes in relapsed or refractory multiple myeloma. Blood Adv..

[23] El-Sherbiny Y.M., Meade J.L., Holmes T.D., McGonagle D., Mackie S.L., Morgan A.W., Cook G., Feyler S., Richards S.J., Davies F.E. (2007). The requirement for DNAM-1, NKG2D, and NKp46 in the natural killer cell-mediated killing of myeloma cells. Cancer Res..

[24] Fauriat C., Mallet F., Olive D., Costello R.T. (2006). Impaired activating receptor expression pattern in natural killer cells from patients with multiple myeloma. Leukemia.

[25] Guillerey C., de Andrade L.F., Vuckovic S., Miles K., Ngiow S.F., Yong M.C., Teng M.W., Colonna M., Ritchie D.S., Chesi M. (2015). Immunosurveillance and therapy of multiple myeloma are CD226 dependent. J. Clin. Investig..

[26] Ponzetta A., Benigni G., Antonangeli F., Sciume G., Sanseviero E., Zingoni A., Ricciardi M.R., Petrucci M.T., Santoni A., Bernardini G. (2015). Multiple myeloma impairs bone marrow localization of effector natural killer cells by altering the chemokine microenvironment. Cancer Res..

[27] Benson D.M., Bakan C.E., Zhang S., Collins S.M., Liang J., Srivastava S., Hofmeister C.C., Efebera Y., Andre P., Romagne F. (2011). IPH2101, a novel anti-inhibitory KIR antibody, and lenalidomide combine to enhance the natural killer cell versus multiple myeloma effect. Blood.

[28] Molfetta R., Quatrini L., Zitti B., Capuano C., Galandrini R., Santoni A., Paolini R. (2016). Regulation of NKG2D expression and signaling by endocytosis. Trends Immunol..

[29] Sabry M., Zubiak A., Hood S.P., Simmonds P., Arellano-Ballestero H., Cournoyer E., Mashar M., Pockley A.G., Lowdell M.W. (2019). Tumor- and cytokine-primed human natural killer cells exhibit distinct phenotypic and transcriptional signatures. PLoS ONE.

[30] Vulpis E., Stabile H., Soriani A., Fionda C., Petrucci M.T., Mariggio E., Ricciardi M.R., Cippitelli M., Gismondi A., Santoni A. (2018). Key role of the CD56(low)CD16(low) natural killer cell subset in the recognition and killing of multiple myeloma cells. Cancers.

[31] Guillamon C.F., Martinez-Sanchez M.V., Gimeno L., Campillo J.A., Server-Pastor G., Martinez-Garcia J., Martinez-Escribano J., Torroba A., Ferri B., Abellan D.J. (2019). Activating KIRs on educated NK cells support downregulation of CD226 and inefficient tumor immunosurveillance. Cancer Immunol. Res..

[32] von Lilienfeld-Toal M., Frank S., Leyendecker C., Feyler S., Jarmin S., Morgan R., Glasmacher A., Marten A., Schmidt-Wolf I.G., Brossart P. (2010). Reduced immune effector cell NKG2D expression and increased levels of soluble NKG2D ligands in multiple myeloma may not be causally linked. Cancer Immunol. Immunother..

[33] Gill S., Vasey A.E., De Souza A., Baker J., Smith A.T., Kohrt H.E., Florek M., Gibbs K.D., Tate K., Ritchie D.S. (2012). Rapid development of exhaustion and down-regulation of eomesodermin limit the antitumor activity of adoptively transferred murine natural killer cells. Blood.

[34] Jinushi M., Vanneman M., Munshi N.C., Tai Y.T., Prabhala R.H., Ritz J., Neuberg D., Anderson K.C., Carrasco D.R., Dranoff G. (2008). MHC class I chain-related protein A antibodies and shedding are associated with the progression of multiple myeloma. Proc. Natl. Acad. Sci. USA.

[35] Niu C., Jin H., Li M., Zhu S., Zhou L., Jin F., Zhou Y., Xu D., Xu J., Zhao L. (2017). Low-dose bortezomib increases the expression of NKG2D and DNAM-1 ligands and enhances induced NK and gammadelta T cell-mediated lysis in multiple myeloma. Oncotarget.

[36] Soriani A., Iannitto M.L., Ricci B., Fionda C., Malgarini G., Morrone S., Peruzzi G., Ricciardi M.R., Petrucci M.T., Cippitelli M. (2014). Reactive oxygen species- and DNA damage response-dependent NK cell activating ligand upregulation occurs at transcriptional levels and requires the transcriptional factor E2F1. J. Immunol..

[37] Fionda C., Abruzzese M.P., Zingoni A., Cecere F., Vulpis E., Peruzzi G., Soriani A., Molfetta R., Paolini R., Ricciardi M.R. (2015). The IMiDs targets IKZF-1/3 and IRF4 as novel negative regulators of NK cell-activating ligands expression in multiple myeloma. Oncotarget.

[38] Frassanito M.A., Silvestris F., Cafforio P., Silvestris N., Dammacco F. (1997). IgG M-components in active myeloma patients induce a down-regulation of natural killer cell activity. Int. J. Clin. Lab. Res..

[39] Jurisic V., Srdic T., Konjevic G., Markovic O., Colovic M. (2007). Clinical stage-depending decrease of NK cell activity in multiple myeloma patients. Med. Oncol..

[40] Schmiedel B.J., Werner A., Steinbacher J., Nuebling T., Buechele C., Grosse-Hovest L., Salih H.R. (2013). Generation and preclinical characterization of a Fc-optimized GITR-Ig fusion protein for induction of NK cell reactivity against leukemia. Mol. Ther..

[41] Buechele C., Baessler T., Wirths S., Schmohl J.U., Schmiedel B.J., Salih H.R. (2012). Glucocorticoid-induced TNFR-related protein (GITR) ligand modulates cytokine release and NK cell reactivity in chronic lymphocytic leukemia (CLL). Leukemia.

[42] Ogasawara K., Yoshinaga S.K., Lanier L.L. (2002). Inducible costimulator costimulates cytotoxic activity and IFN-gamma production in activated murine NK cells. J. Immunol..

[43] Rischin D., Groenland S.L., Lim A.M.L., Martin-Liberal J., Moreno V., Perez J.M.T., Le Tourneau C., Mathew M., Cho D.C., Hansen A.R. (2019). Inducible T cell costimulatory (ICOS) receptor agonist, GSK3359609 (GSK609) alone and in combination with pembrolizumab (pembro): Preliminary results from INDUCE-1 expansion cohorts (EC) in head and neck squamous cell carcinoma (HNSCC). Ann. Oncol..

[44] Kim N., Kim H.S. (2018). Targeting checkpoint receptors and molecules for therapeutic modulation of natural killer cells. Front. Immunol..

[45] Guillerey C., Harjunpaa H., Carrie N., Kassem S., Teo T., Miles K., Krumeich S., Weulersse M., Cuisinier M., Stannard K. (2018). TIGIT immune checkpoint blockade restores CD8(+) T-cell immunity against multiple myeloma. Blood.

[46] Minnie S.A., Kuns R.D., Gartlan K.H., Zhang P., Wilkinson A.N., Samson L., Guillerey C., Engwerda C., MacDonald K.P.A., Smyth M.J. (2018). Myeloma escape after stem cell transplantation is a consequence of T-cell exhaustion and is prevented by TIGIT blockade. Blood.

[47] Stenner F., Renner C. (2018). Cancer immunotherapy and the immune response in follicular lymphoma. Front. Oncol..

[48] Zhang J., Medeiros L.J., Young K.H. (2018). Cancer immunotherapy in diffuse large B-cell lymphoma. Front. Oncol..

[49] Lesokhin A.M., Bal S., Badros A.Z. (2019). Lessons learned from checkpoint blockade targeting PD-1 in multiple myeloma. Cancer Immunol. Res..

[50] Cohen A.D. (2019). Myeloma: Next generation immunotherapy. Hematol. Am. Soc. Hematol. Educ. Program..

[51] Pazina T., James A.M., Colby K.B., Yang Y., Gale A., Jhatakia A., Kearney A.Y., Graziano R.F., Bezman N.A., Robbins M.D. (2019). Elotuzumab enhances SLAMF7 interactions between natural killer and multiple myeloma cells to co-stimulate cytotoxicity. Cancer Immunol. Res..

[52] Pazina T., James A.M., MacFarlane A.W., Bezman N.A., Henning K.A., Bee C., Graziano R.F., Robbins M.D., Cohen A.D., Campbell K.S. (2017). The anti-SLAMF7 antibody elotuzumab mediates NK cell activation through both CD16-dependent and -independent mechanisms. Oncoimmunology.

[53] Sundararajan S., Kumar A., Korde N., Agarwal A. (2016). Smoldering multiple myeloma: Emerging concepts and therapeutics. Curr. Hematol. Malig. Rep..

[54] MacFarlane A.W., Jillab M., Smith M.R., Alpaugh R.K., Cole M.E., Litwin S., Millenson M.M., Al-Saleem T., Cohen A.D., Campbell K.S. (2017). NK cell dysfunction in chronic lymphocytic leukemia is associated with loss of the mature cells expressing inhibitory killer cell Ig-like receptors. Oncoimmunology.

[55] MacFarlane A.W., Jillab M., Plimack E.R., Hudes G.R., Uzzo R.G., Litwin S., Dulaimi E., Al-Saleem T., Campbell K.S. (2014). PD-1 expression on peripheral blood cells increases with stage in renal cell carcinoma patients and is rapidly reduced after surgical tumor resection. Cancer Immunol. Res..

