# Supplementary material for: Alterations of NK Cell Phenotype in the Disease Course of Multiple Myeloma
Source: Cancers (Basel). 2021 Jan 10;13(2):226. doi: 10.3390/cancers13020226 (PMC7827733; doi:10.3390/cancers13020226)
Supplement: Supplementary file 1 [file cancers-13-00226-s001.pdf]

cancers

MDPI

Article

Alterations of NK Cell Phenotype in the Disease Course of Multiple Myeloma

Tatiana Pazina, Alexander W. MacFarlane IV, Luca Bernabei, Essel Dulaimi, Rebecca Kotcher, Clinton Yam, Natalie A. Bezman, Michael D. Robbins, Eric A. Ross, Kerry S. Campbell and Adam D. Cohen

Supplementary Material

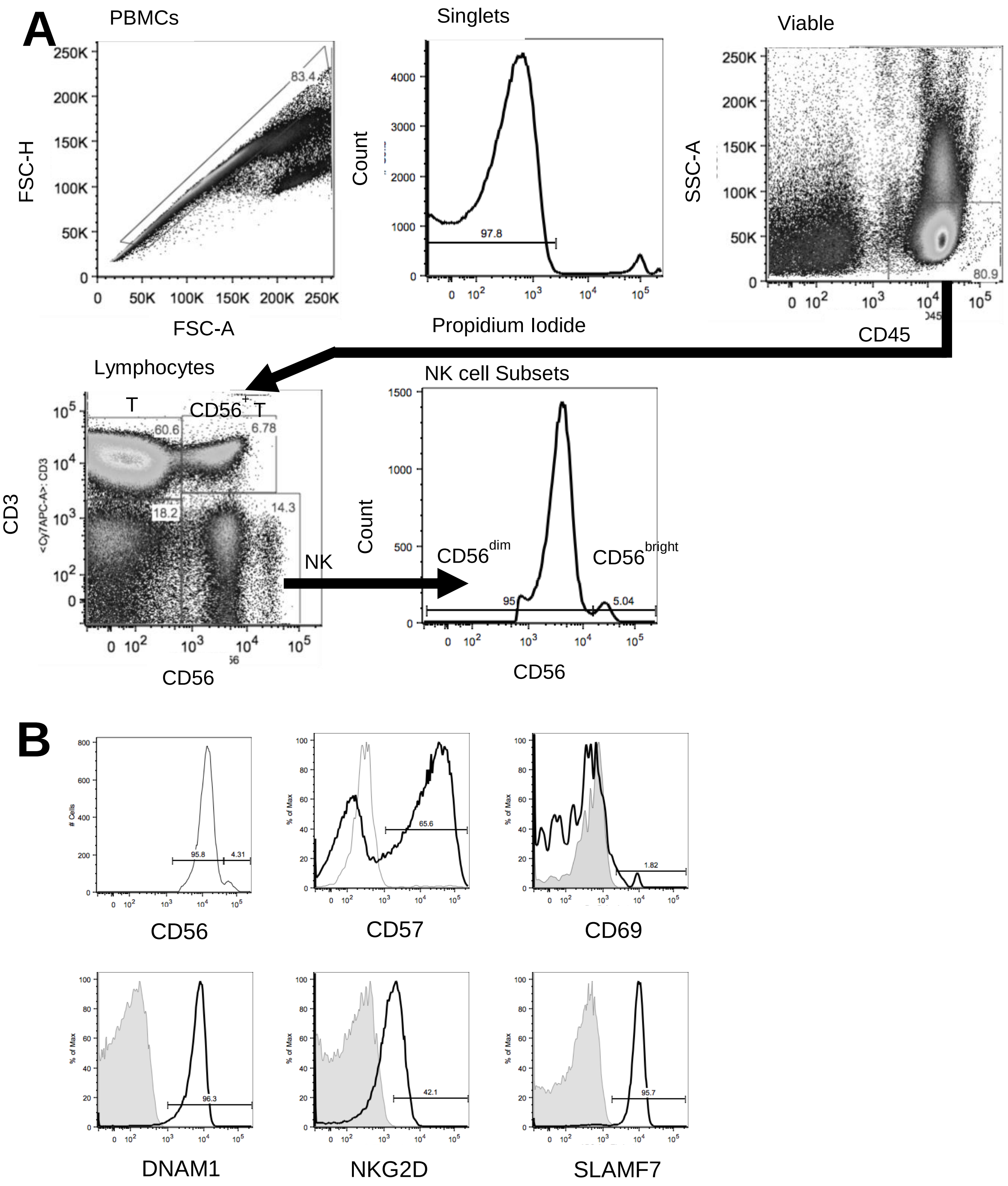

Figure S1. Representative gating and staining of NK cells. (**A**) Gating. strategy. PBMCs from a MM patient were stained for 20 min on ice. Gates were applied to select single cells, propidium-negative viable cells, lymphocytes and then lymphocyte subsets.NK cell subsets were further divided into CD45^{+}CD3^{-}CD56^{bright} and CD56^{dim} NK cells. (**B**) Representative staining of CD56^{bright} vs CD56^{dim} NK cells and percentage of cells staining forpositive expression of CD57, CD69, DNAM1, NKG2D, and SLAMF7 on NK cells from blood. Gates for % positive cells were set where <1% of cells were gated in isotype control or.unstained control samples (grey).

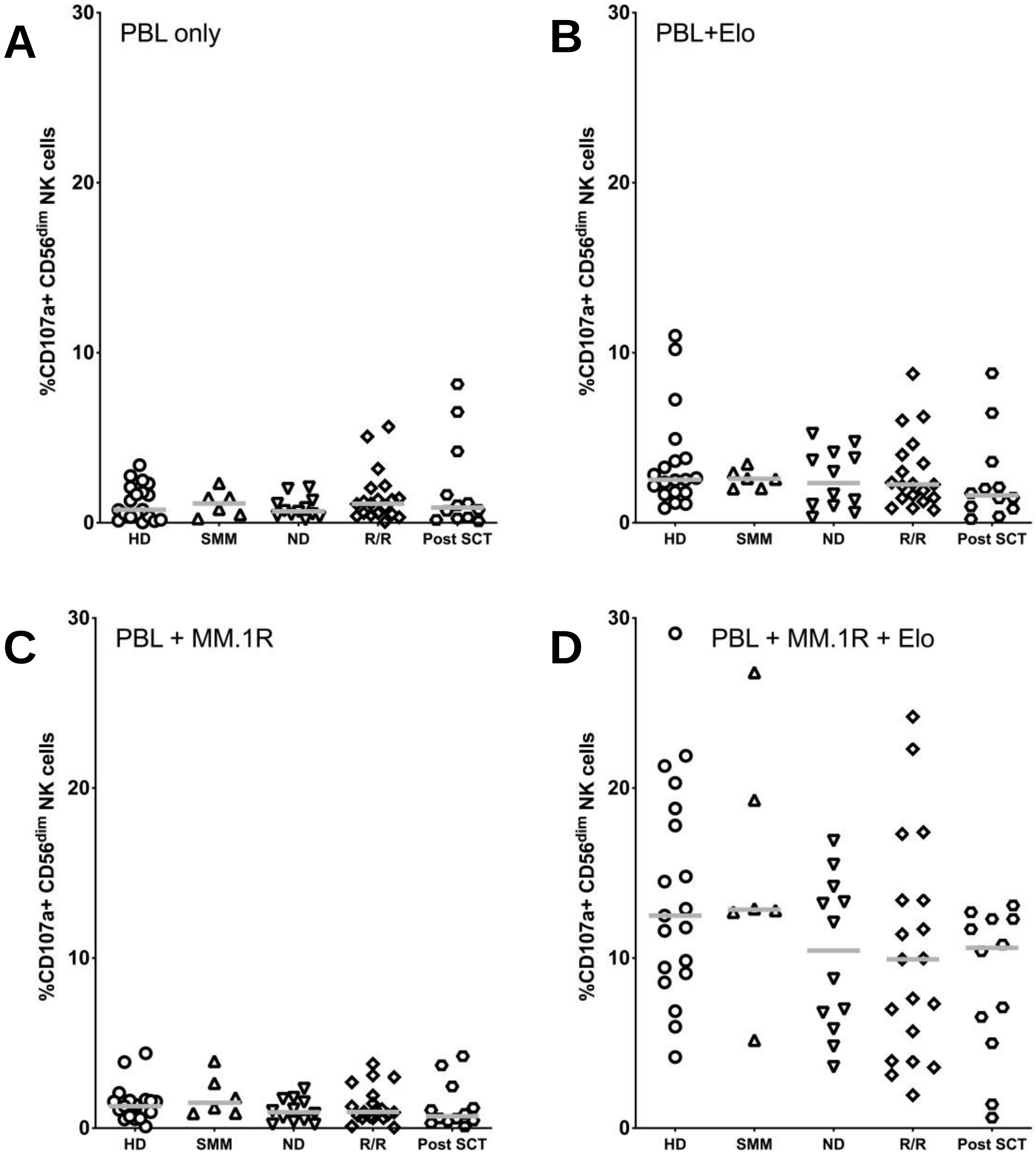

Figure S2. Effects of elotuzumab on NK cell degranulation. PBMCs were incubated alone, with elotuzumab (Elo), with MM.1R target cells, or with target cells and Elo for 2 h at 37 °C and stained for percent LAMP-1 (CD107a)$^+$ cells. NK cells were gated as CD45$^+$CD3$^-$CD56^{dim} cells after the viability gate. Horizontal lines mark median values, and statistics were calculated with unpaired Mann-Whitney test.

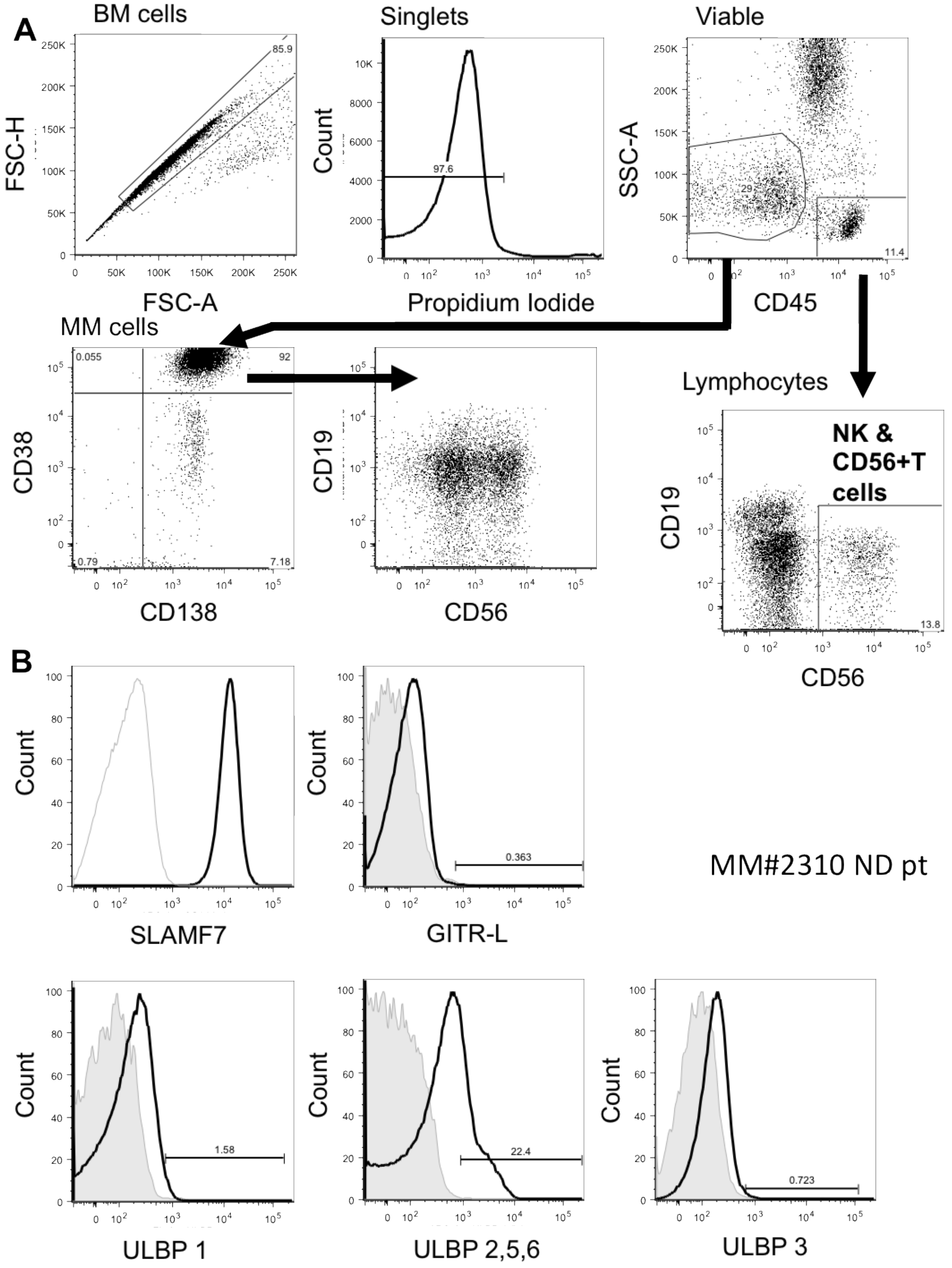

Figure S3. BM gating. (**A**) BM cells were purified from BM aspirates of ND patient and stained for 20 min on ice. Gates were applied to select single cells, propidium-negative viable cells, MM tumor cells (left box gate) and lymphocytes (right box gate). MM cells were identified as $CD45^{dim}$, $CD38^{++}$, $CD138^{+}$, $CD56^{+/-}$, $CD19^{+/-}$. Lymphocytes subsets were further divided into $CD19^{-}CD56^{+}$ NK and $CD56^{+}$ T cells that were used as negative population for GITR-L, ULBPs staining in B. (**B**) Example of SLAMF7, GITR-L, ULBP 1, ULBP 2,5,6, ULBP 3 staining on viable MM cells gated in (**A**). For SLAMF7 staining unstained cells are shown in grey.

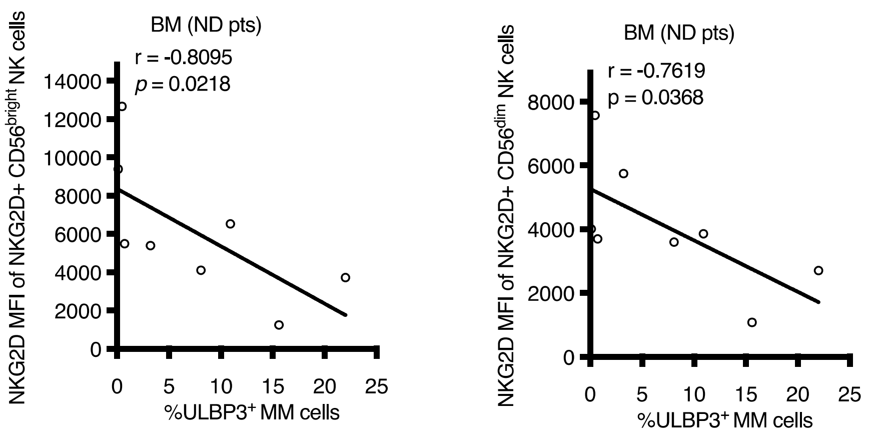

Figure S4. Correlation of NKG2D expression on BM NK cells with ULBP3 ligand expression on MM cells in BM of ND patients. Each symbol is an individual patient. Statistics were calculated with Spearman's rank correlation coefficient, lines are linear least squares fit for visual purposes only. Comparisons are of MFI of NKG2D expression on $CD56^{bright}$ (left) or $CD56^{dim}$ (right) blood NK cell populations vs. % MM cells in BM that are $ULBP3^{+}$.

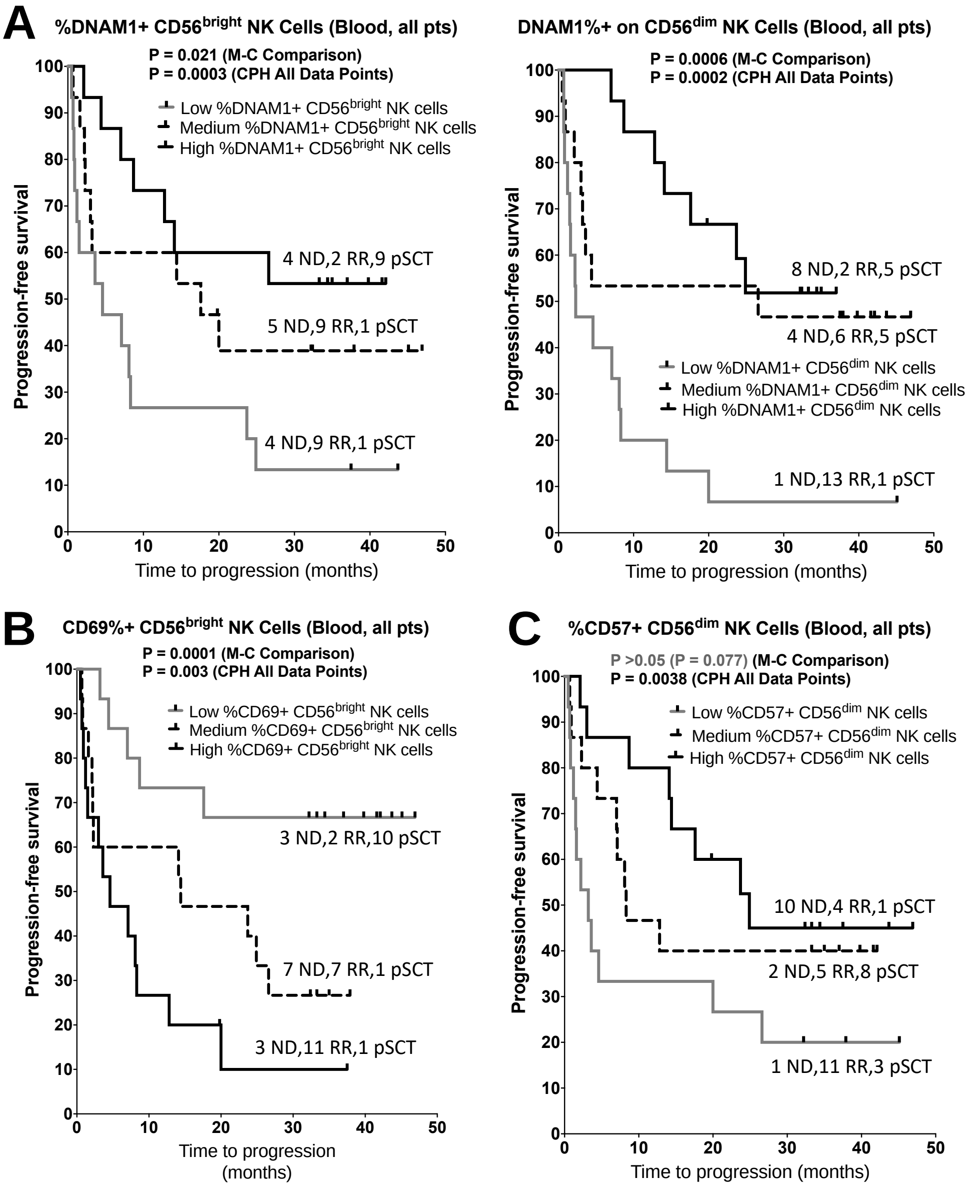

Figure S5. Kaplan-Meier survival plots showing time to progression based on expression of various NK cell markers for the combined ND, RR, and pSCT MM patients divided into tertiles. Patients were divided into tertiles based on their (**A**) % CD56^{bright} and CD56^{dim} NK cells that are DNAM1^{+}, (**B**) % CD56^{bright} NK cells that are CD69^{+}, and (**C**) % CD56^{dim} NK cells that are CD57^{+} in blood. Dark solid lines represent high expression tertile, dashed dark lines designate medium expression tertile, and solid grey line is low expression tertile for each surface marker. M-C = Mantel-Cox statistical analysis of tertiles, CPH = Cox-Proportional Hazards test performed on all data points. Numbers of patients in each tertile that are newly diagnosed (ND), relapsed/refractory (RR), or post stem cell transplant (pSCT) are indicated adjacent to each plotted line.

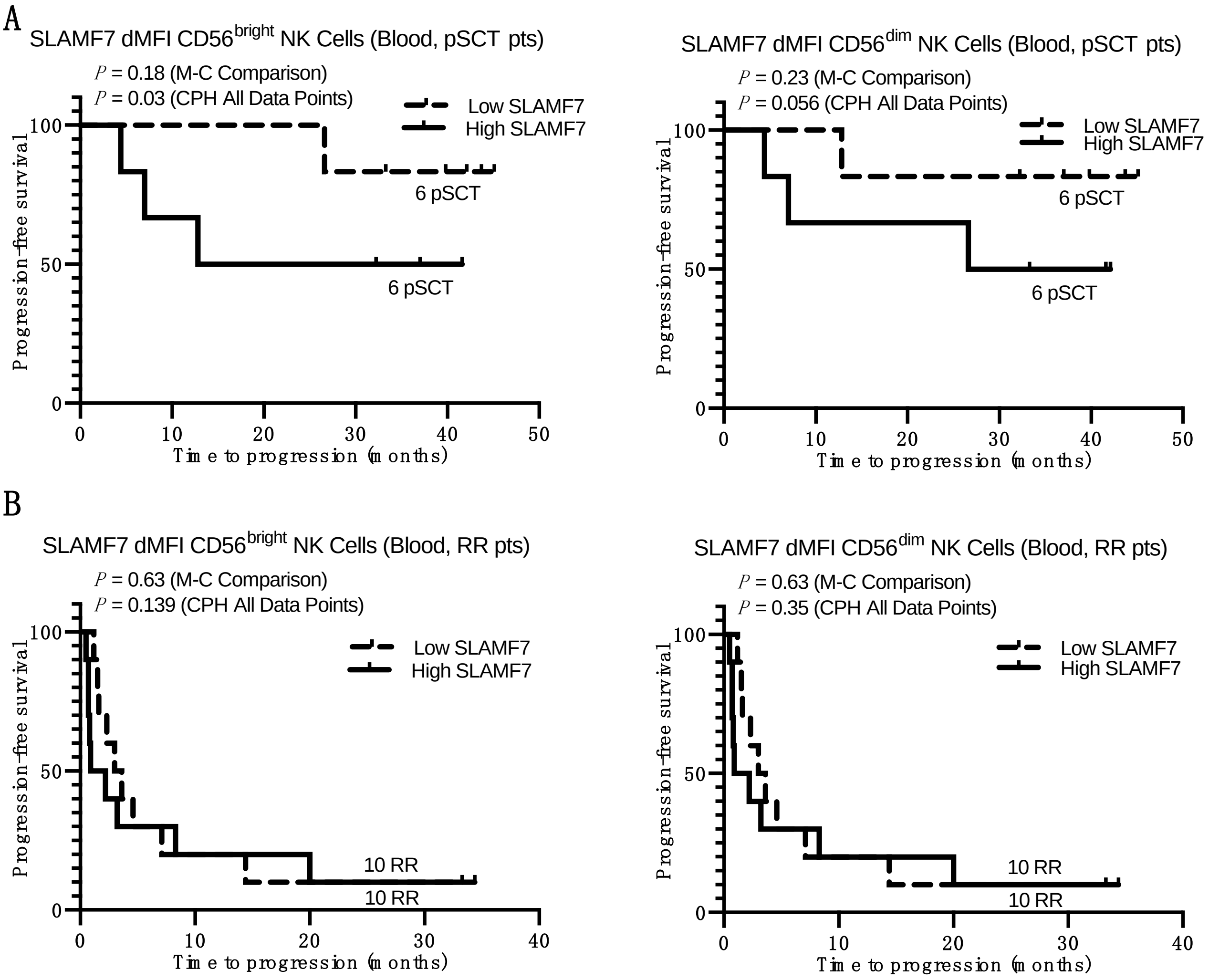

Figure S6. Kaplan-Meier survival plots showing time to progression as a function of SLAMF7 expression on blood NK cells in pSCT and RR MM patients. The pSCT (**A**) and RR (**B**) patients were divided into halves above and below median based on the dMFI of SLAMF7 expression on their CD56^{bright} (left) and CD56^{dim} NK cells (right) in peripheral blood. Solid lines designate the upper half and dashed lines are the lower half of SLAMF7 expression. M-C = Mantel-Cox statistical analysis of halves, CPH = Cox-Proportional Hazards test performed on all data points.

Table S1. NK cell antibody staining panel for flow cytometry. Each column represents one staining tube and each row is a fluorescent channel on the flow cytometer. Monoclonal antibodies to markers are listed, followed by clone name and manufacturer. (EB = eBioscience, BL = Biolegend, BD = BD Pharmingen, BC = Beckman Coulter, BMS = Bristol-Myers Squibb).

Fluorophore	NK #1	NK #2	NK #3	T/NK	T	Isotype #1	Isotype #2	Isotype #3
**FITC**	CD19 H1B19 (EB)	CD56 NCAM 16.2 (BD)	KIR2DL1/KIR2DS1 EB6B (BC)	CD56 NCAM 16.2 (BD)	CD25 2A3 (EB)	CD56 NCAM 16.2 (BD)	CD56 NCAM 16.2 (BD)	CD56 NCAM 16.2 (BD)
**PE**	CD57 NK1 (BD)	KIR3DL1/ KIR3DS1 Z27.3.7 (BC)	KIR2DL1/CD158a 143211 (R&D)	CD4 OKT4 (BL)	CD62L DREG-56 (BL)			
**APC**	SLAMF7 Elo-tuzumab (BMS)	DNAM-1 11A8 (BL)	KIR2DS4 FES172 (BC)	TIGIT MBSA43 (EB)	PD-1 MIH4 (EB)	huIgG1-κ (Southern Biotech)	mIgG1-κ (EB)	
**PerCP/ Cy5.5**	CD45 2D1 (EB)	CD45 2D1 (EB)	CD45 2D1 (EB)	LAG3 3DS223H (EB)	CD45RA HI100 (BL)	CD45 2D1 (EB)	CD45 2D1 (EB)	CD45 2D1 (EB)
**Cy7/APC**	CD3 SK7 (BD)	CD3 SK7 (BD)	CD3 SK7 (BD)	CD3 SK7 (BD)	CD3 SK7 (BD)	CD3 SK7 (BD)	CD3 SK7 (BD)	CD3 SK7 (BD)
**Alexa-Fluor 700**	CD137 4B4-1 (BL)	KIR3DL1 DX9 (BL)	CD11a MEM25 (Exbio)	CD8 HIT8a (BL)	CD8 HIT8a (BL)	CD16 3G8 (BL)	mIgG1-κ (BL)	
**QDot 605**		NKp44 P44-8 (BL)	KLRG1 2F1/KLRG1 (BL)	TIM3 F38-2E2 (BL)	CD127 eBioRDR5 (EB)		mIgG1-κ (BL)	Syrian Ham. IgG (BL)
**Cy7/PE**	CD56 NCAM 16.2 (BD)	NKp30 AF29-4D12 (EB)	CD56 NCAM 16.2 (BD)	OX40 Ber-ACT35 (BL)	CD56 NCAM 16.2 (BD)	NKG2D 1D11 (BL)	mIgG1-κ (BL)	
**PerCP**	Propidium Iodide	Propidium Iodide	Propidium Iodide	Propidium Iodide	Propidium Iodide	Propidium Iodide	Propidium Iodide	Propidium Iodide
**Pacific Blue**	CD69 (FN50) (BL)	GITR eBioAITR (EB)	NKp46 9E2 (EB)	ICOS C398.4A (BL)	CD4 OKT4 (BL)	CD19 HIB19 (BD)	mIgG1-κ (BL)	Arm. Ham. IgG (BL)
**Brilliant Violet 510**			PD-1 EH12.2H7 (BL)	PD-1 EH12.2H7 (BL)	CD28 CD28.2 (BL)		mIgG1-κ (BL)	

Table S2. Ligands on MM cells antibody staining panel for BM samples assessed by flow cytometry. Each column represents one staining tube and each row is a fluorescent channel on the flow cytometer. Monoclonal antibodies to markers are listed, followed by clone name and manufacturer. (EB = eBioscience, BL = Biolegend, BD = BD Pharmingen, BC = Beckman Coulter, BMS = Bristol-Myers Squibb, RD = R&D Systems).

Fluorophore	#1	#2	#3	#4
**FITC**	CD56<br>NCAM 16.2<br>(BD)	HLA-A,B,C<br>W6/32<br>(BL)	CD56<br>NCAM 16.2<br>(BD)	ULBP1<br>FAB1380G<br>(RD)
**PE**	CD137-L<br>5F4<br>(BL)	Nectin 2 (CD112)<br>TX3<br>(BL)	GITR-L<br>109101<br>(RD)	ULBP3<br>FAB1517P<br>(RD)
**APC**	MICA/MICB<br>6D4<br>(BL)	PVR (CD155)<br>300907<br>(RD)	SLAMF7<br>Elotuzumab<br>(BMS)	ULBP2,5,6<br>FAB1298A<br>(RD)
**PerCP/Cy5.5**	CD45<br>2D1<br>(EB)	CD45<br>2D1<br>(EB)	CD45<br>2D1<br>(EB)	CD45<br>2D1<br>(EB)
**Cy7/APC**	CD38<br>HIT2<br>(BL)	CD38<br>HIT2<br>(BL)	CD38<br>HIT2<br>(BL)	CD38<br>HIT2<br>(BL)
**AlexaFluor 700**	CD19<br>H1B19<br>(EB)	CD19<br>H1B19<br>(EB)	CD19<br>H1B19<br>(EB)	CD19<br>H1B19<br>(EB)
**QDot 605**	PDL2<br>MIH18<br>(EB)	ICOS-L<br>MIH12<br>(EB)		BCMA<br>BAF193<br>(RD)
**Cy7/PE**	PDL1<br>29E2A3<br>(BL)	CD56<br>NCAM 16.2<br>(BD)		CD56<br>NCAM 16.2<br>(BD)
**PerCP**	Propidium<br>Iodide	Propidium<br>Iodide	Propidium<br>Iodide	Propidium<br>Iodide
**Pacific Blue**	CD138<br>MI15<br>(BD)	CD138<br>MI15<br>(BD)	CD138<br>MI15<br>(BD)	CD138<br>MI15<br>(BD)
